# UV radiation‐induced peptides in frog skin confer protection against cutaneous photodamage through suppressing MAPK signaling

**DOI:** 10.1002/mco2.625

**Published:** 2024-06-25

**Authors:** Tingyi Yang, Fenghao Geng, Xiaoyou Tang, Zuxiang Yu, Yulan Liu, Bin Song, Zhihui Tang, Baoning Wang, Bengui Ye, Daojiang Yu, Shuyu Zhang

**Affiliations:** ^1^ Laboratory of Radiation Medicine West China School of Basic Medical Sciences & Forensic Medicine Sichuan University Chengdu China; ^2^ Medical College of Tibet University, Tibet University Lhasa China; ^3^ The Second Affiliated Hospital of Chengdu Medical College China National Nuclear Corporation 416 Hospital Chengdu China; ^4^ NHC Key Laboratory of Nuclear Technology Medical Transformation (Mianyang Central Hospital) Mianyang China

**Keywords:** frog, photodamage, skin, ultraviolet (UV) radiation, ultraviolet B (UVB)

## Abstract

Overexposure to ultraviolet light (UV) has become a major dermatological problem since the intensity of ultraviolet radiation is increasing. As an adaption to outside environments, amphibians gained an excellent peptide‐based defense system in their naked skin from secular evolution. Here, we first determined the adaptation and resistance of the dark‐spotted frogs *(Pelophylax nigromaculatus*) to constant ultraviolet B (UVB) exposure. Subsequently, peptidomics of frog skin identified a series of novel peptides in response to UVB. These UV‐induced frog skin peptides (UIFSPs) conferred significant protection against UVB‐induced death and senescence in skin cells. Moreover, the protective effects of UIFSPs were boosted by coupling with the transcription trans‐activating (TAT) protein transduction domain. In vivo, TAT‐conjugated UIFSPs mitigated skin photodamage and accelerated wound healing. Transcriptomic profiling revealed that multiple pathways were modulated by TAT‐conjugated UIFSPs, including small GTPase/Ras signaling and MAPK signaling. Importantly, pharmacological activation of MAPK kinases counteracted UIFSP‐induced decrease in cell death after UVB exposure. Taken together, our findings provide evidence for the potential preventive and therapeutic significance of UIFSPs in UV‐induced skin damage by antagonizing MAPK signaling pathways. In addition, these results suggest a practicable alternative in which potential therapeutic agents can be mined from organisms with a fascinating ability to adapt.

## Graphical abstract

We found a series of UV‐induced frog skin peptides (UIFSPs) from *P. nigromaculatus*. These peptides conferred significant protection against UVB‐induced skin damage, and the photo‐protective efficacy of UIFSPs was enhanced by UIFSPs modification with TAT protein transduction domain. Mechanistically, TAT‐conjugated UIFSPs internalized into skin cells and suppressed UVB‐induced activation of Ras/PI3K/AKT and Ras/MAPKs pathways, thereby preventing skin photodamage.

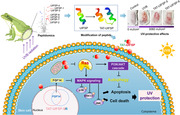

## 1 INTRODUCTION

As the first‐line barrier between internal body and outside environment, skin is the most exposed organ which frequently experiences direct effects of environmental factors, especially exposed to ultraviolet (UV) light on a daily basis.^1^ UV radiation present in sunlight is considered among the most dangerous environmental factors, and the toxic effects of UV from natural sunlight and therapeutic artificial lamps are a major concern for human health.^2^, ^3^ There are three kinds of UV rays, longwave UVA (315–400 nm), medium‐wave UVB (280–315 nm), and shortwave UVC (100–280 nm). In contrast with UVC, which is absorbed efficiently in the atmosphere by ozone and oxygen, the quantity of UVA and UVB radiation that reaches the Earth's surface is sufficient to cause important biological consequences for the skin and eyes.

Compared with UVA, UVB is far more energetic and carcinogenic.^4^ UVB penetrates the epidermis and upper dermis and exerts profound effects on skin.^5^ It has been well reported that UVB radiation alters skin homeostasis, leads to acute inflammation, erythema, skin photoaging, skin pigmentation, and photocarcinogenesis.^6^ Broadly, UV radiation induces reactive oxygen species (ROS) generation by disrupting cellular components directly or by means of photosensitization mechanisms. Skin contains several major UV absorbing endogenous chromophores. Molecules or regions of molecules absorb UVB to become electronically excited and then transfer their energy or donate an electron to O_2_ to form several ROS.^7^, ^8^ When skin homeostasis is disrupted due to ROS generation exceeding the antioxidant defense ability, oxidative stress can develop and drive the process of cellular damage (e.g., lipid peroxidation and DNA fragmentation), apoptosis, and cell death. Moreover, various signaling pathways are activated by UV‐mediated ROS generation, especially in the pathophysiology of skin diseases.

In the struggle for survival of living organisms, they adapt to diverse environmental factors and evolve with advantageous mutations or traits to withstand such challenges and be selected for survival. Amphibians are of particular biological interest because of their extreme chemical diversity, and their skin has evolved a unique and highly effective polypeptide defense system to combat harsh environments.^9^ Various antioxidant peptides (AOPs) have been identified from frog skin secretions, and only a few AOPs exert potency to protect epidermal cells from UVB damage.^10^, ^11^ In addition, peptidomic analysis of skin secretions from two frog species in plateau areas and caves revealed that greater diversity and free radical scavenging potentiality of skin antioxidant peptides evolved with a long duration of sunshine and strong UV radiation.^12^ Studies have shown that tree frogs living within the Chornobyl exclusion zone exhibited a remarkably darker dorsal skin coloration than that of frogs outside the zone, particularly melanin pigmentation acted as a buffering mechanism against ionizing radiation through neutralizing free radical.^13^

In this study, we sought to investigate whether short‐term changes in frogs enable them to adapt to a new ecological niche. The dark‐spotted frog (*Pelophylax nigromaculatus*) is known from the Russian Far East, central, northern and northeastern China, the Korean Peninsula, and part of Japan. It is widely from the frigid belt to the tropical zone and from the plateau to the basin.^14^ The extensive distribution of *P. nigromaculatus* indicates its powerful viability and ability to adapt to various environments. Moreover, this frog species presents a discrepancy in peptide expression profiles between populations from two regions of the same species from Kunming and Guiyang.^15^ To date, only several antimicrobial peptides and antioxidant peptides have been reported from dark‐spotted frogs.^16^ Given the ancient practice of frog for traditional Chinese medicine utilization, particularly the oviduct and skin have been developed into various wholesome products. Therefore, the frog skin may contain the valuable medicinal ingredients.

There is a hypothesis proposing adaptation insight into a targeted mining strategy of radioprotective agents. In our previous work, we found that 30 Gy electron beams altered the endogenous substances in frog skin tissues and induced RIFSP‐2 expression in which RIFSP‐2 serves as a promising radiation mitigator.^17^ Herein, we established an inducibility model of *P. nigromaculatus* by repetitive UVB exposure and observed the physiological adaptations in frogs in response to UVB. By virtue of the peptidomic analysis, we analyzed the molecular basis of UV adaptation and identified the promising pharmacological peptides, which are named as UV‐induced frog skin peptides (UIFSPs). Their role in the photoprotection of human skin cells and animal models was further characterized.

## 2 RESULTS

### 2.1 Inducibility and potential radiation‐responsive substance identification of *P. nigromaculatus* skin to UVB

**FIGURE 1:**
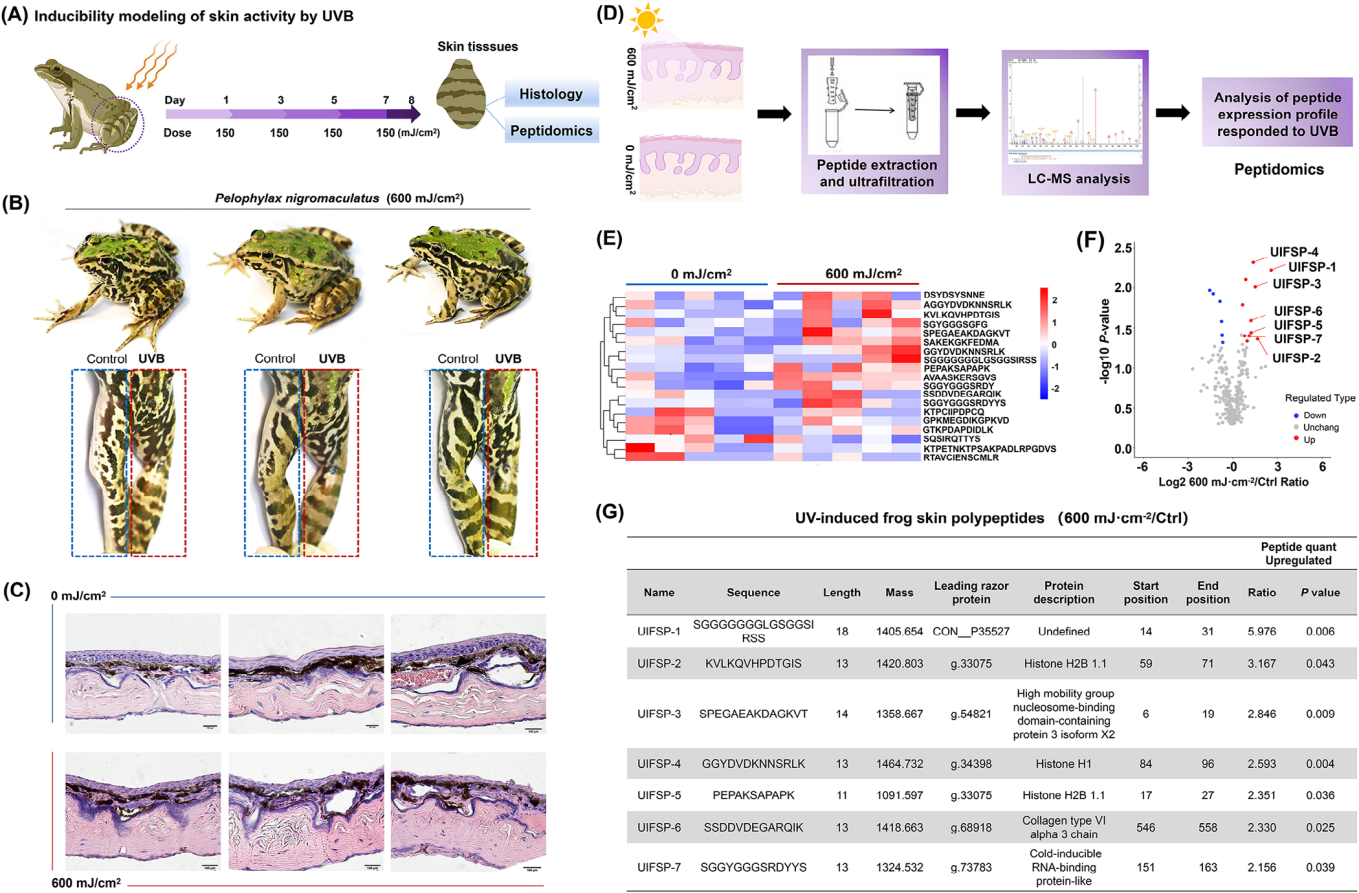
Peptide profiling analysis of skin adaptation response of *P. nigromaculatus* induced by ultraviolet B (UVB) radiation. (A) Flowchart of experimental design for the inducibility modeling of frog skin with continuous UVB exposure four times by a single dose of 150 mJ/cm^2^ and a total of 600 mJ/cm^2^. (B) Representative images and (C) H&E staining of frog skin 24‐h post UVB exposure (600 mJ/cm^2^) (scale bar = 100 µm), *n *= 5. (D) Flowchart of quantitative peptidomic analysis from skin samples of 600 mJ/cm^2^. (E) Heatmap and (F) volcano plot of differentially expressed genes. (G) Table of the top seven upregulated peptides in UVB‐exposed frog skin.

As organisms are endowed by natural selection with highly specific and diversified bioactive components that target key physiological elements,^18^ we explored whether *P. nigromaculatus*, frog with powerful viability and strong adaptability, could be developed as promising repository for the bioactive substances' discovery. First, we explored the adaptative response of frog skin to UVB via an inducibility model by constant UVB exposure. After 600 mJ/cm^2^ exposure, consisting of 150 mJ/cm^2^ exposure per day for irradiation every other day (Figure 1A), *P. nigromaculatus* skin retained structural integrity with no obvious skin damage (Figure 1B). Furthermore, histological analysis indicated that the UVB‐exposed skin maintained a compact and well‐stratified structure, retaining the integrity of epidermis and dermis. However, it exhibited a notable increase in melanocyte count and glandular activity, indicative of an adaptive response to the UVB exposure (Figure 1C). To unravel molecular basis for the skin reaction to UVB exposure, we performed peptidomic analysis because it has been reported that amphibian skin chemical defense system was composed of gene‐encoded peptides/proteins. An important point that peptide extraction of skin tissues was prepared with native structure through 10 kDa molecular weight cut‐off (MWCO) filters (Figure 1D).

After 600 mJ/cm^2^ UVB exposure, the polypeptide expression profile of frog skin mainly changed the increase in some peptide contents (Figure 1E). We identified 19 fractions that were differentially expressed after UVB exposure, with 13 upregulated polypeptides and six downregulated polypeptides with a 1.2‐fold change (Figure 1F). These novel, unreported components were generated from UVB‐induced skin activity, thus we named UIFSPs (the information was listed in Tables S4 and S5). Furthermore, through integrative analysis of peptidomics data and the RNA‐Seq data of *P. nigromaculatus* skin, UIFSPs were regarded as fragments mainly from structural proteins, such as collagen or mainly histones (Figure 1G), and their MS/MS spectra are shown in Figure S1. Because UIFSP‐6 derived from extracellular matrix protein (g.68918 Collagen type VI alpha 3 chain), we predicted the potential cleavage sites for UIFSPs sequence through PROSPER and the possibility of UIFSPs as signal peptides by SignalP 5.0, whereas all the seven UIFSP sequences were not signal peptide (Figure S2). It is well known the endogenous peptides are low abundance but with high activity, all in all, the peptide production from stress response seems to be an efficient strategy for frog to combat UV energy. To ascertain their bioactivity, seven upregulated peptides with twofold changes were synthesized for the function test in vivo and in vitro.

### 2.2 Antioxidant properties of UIFSPs protect against UVB‐induced oxidative stress

**FIGURE 2:**
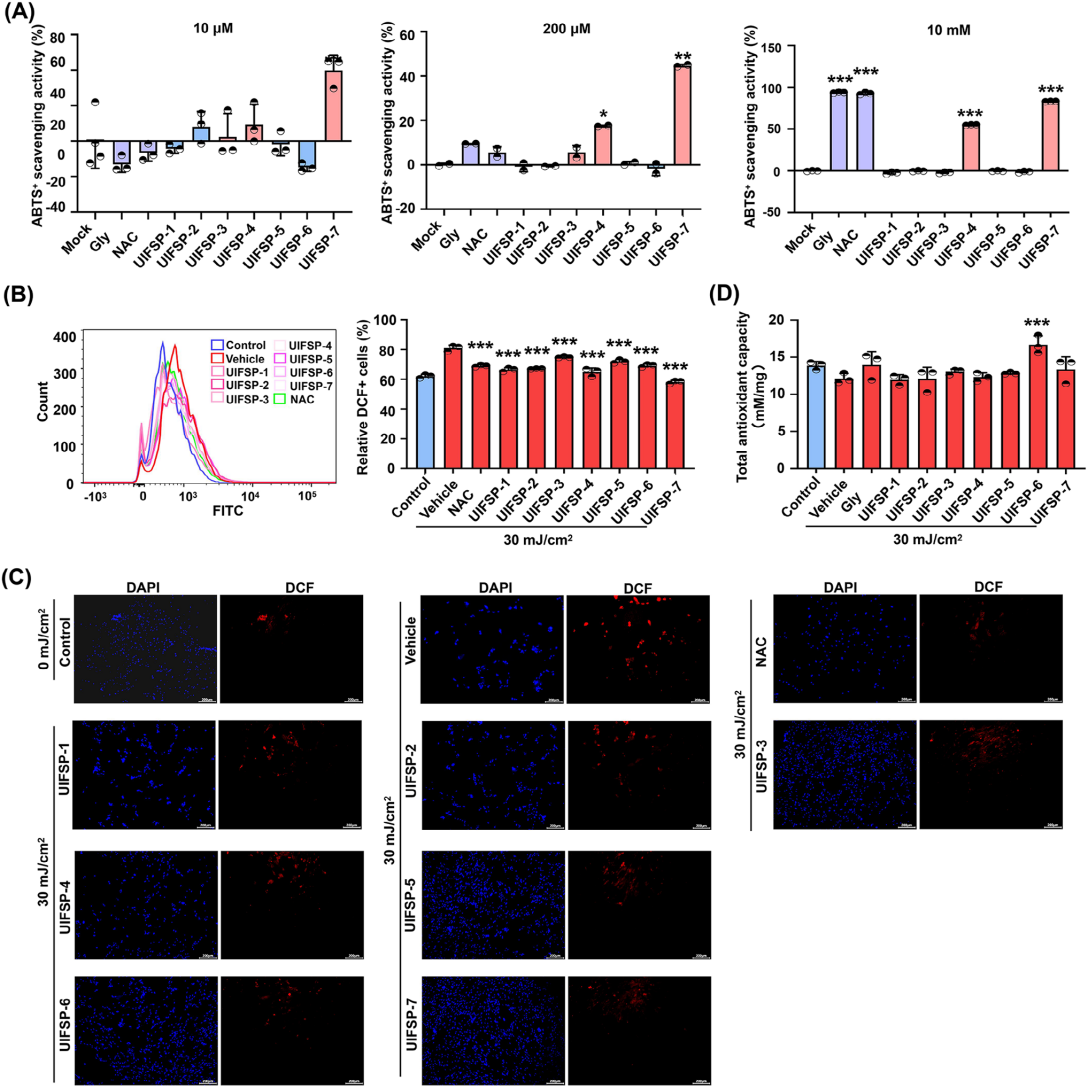
UV‐induced frog skin peptides (UIFSPs) reduce free radicals and improved the antioxidant defense system in ultraviolet B (UVB)‐exposed skin cells. (A) ABTS^+^ radical scavenging activity of UIFSPs at concentrations of 10 µM, 200 µM, and 10 mM. UIFSPs, glycine, and NAC were dissolved in deionized water and mixed with 2,2′‐azino‐bis (3‐ethylbenzothiazoline‐6‐sulfonic acid) (ABTS) solution to measure the absorbance at 415 nm, *n *= 3. The effect of UIFSPs on (B) and (C) ROS levels in WS1 cells through flow cytometry and fluorescence, *n *= 3. (D) Total antioxidant capacity of UIFSPs in WS1 cells, *n *= 3. Cells were pretreated with UIFSPs, glycine, or N‐acetylcysteine (NAC) (20 µM) and exposed to UVB (30 mJ/cm^2^). After 24 h, the ROS levels were detected by DCFH‐DA staining and TCA levels by ABTS agents incubating. Student's *t‐*test and one‐way analysis of variance (ANOVA) were used to evaluate the differences between groups. *p < 0.05, **p < 0.01, and ***p < 0.001, compared to the mock or vehicle group.

Because the natural biopeptides are commonly known to have significant antioxidative and antimicrobial ability, we used the 2,2′‐azino‐bis (3‐ethylbenzothiazoline‐6‐sulfonic acid) (ABTS) assay to examine the radical scavenging capacity of UIFSPs. As shown in Figure 2A, UIFSPs exhibited a dose‐dependent radical scavenging capacity. Specifically, compared with positive control, N‐acetylcysteine (NAC), and Gly, only UIFSP‐7 worked at a lower concentration of 10 µM and both UIFSP‐4 and UIFSP‐7 scavenged at 200 µM, whereas NAC and Gly showed no radical scavenging capacity. At the concentration of 10 mM, both NAC and Gly, and UIFSP‐4 and ‐7 exhibited obvious antioxidant properties. As the significant direct radical scavenging activity of UIFSPs, we next detected their effects on the intracellular UV‐induced ROS overproduction. Exposure to UVB significantly promoted ROS production (Figure 2B,C), whereas all UIFSPs inhibited production of UVB‐induced ROS in WS1 cells at the concentration of 20 µM. Despite cellular antioxidant systems was not significantly decreased after UVB exposure, UIFSP‐6 remarkably improved the antioxidant capacity of UVB‐irradiated skin cells (Figure 2D). However, UIFSPs had no potency against pathogens (Figure S3 and Table S5). These results implied that UIFSPs had a greater impact on UVB‐induced oxidative stress.

### 2.3 UIFSPs confer protection against UVB‐induced skin damage

**FIGURE 3:**
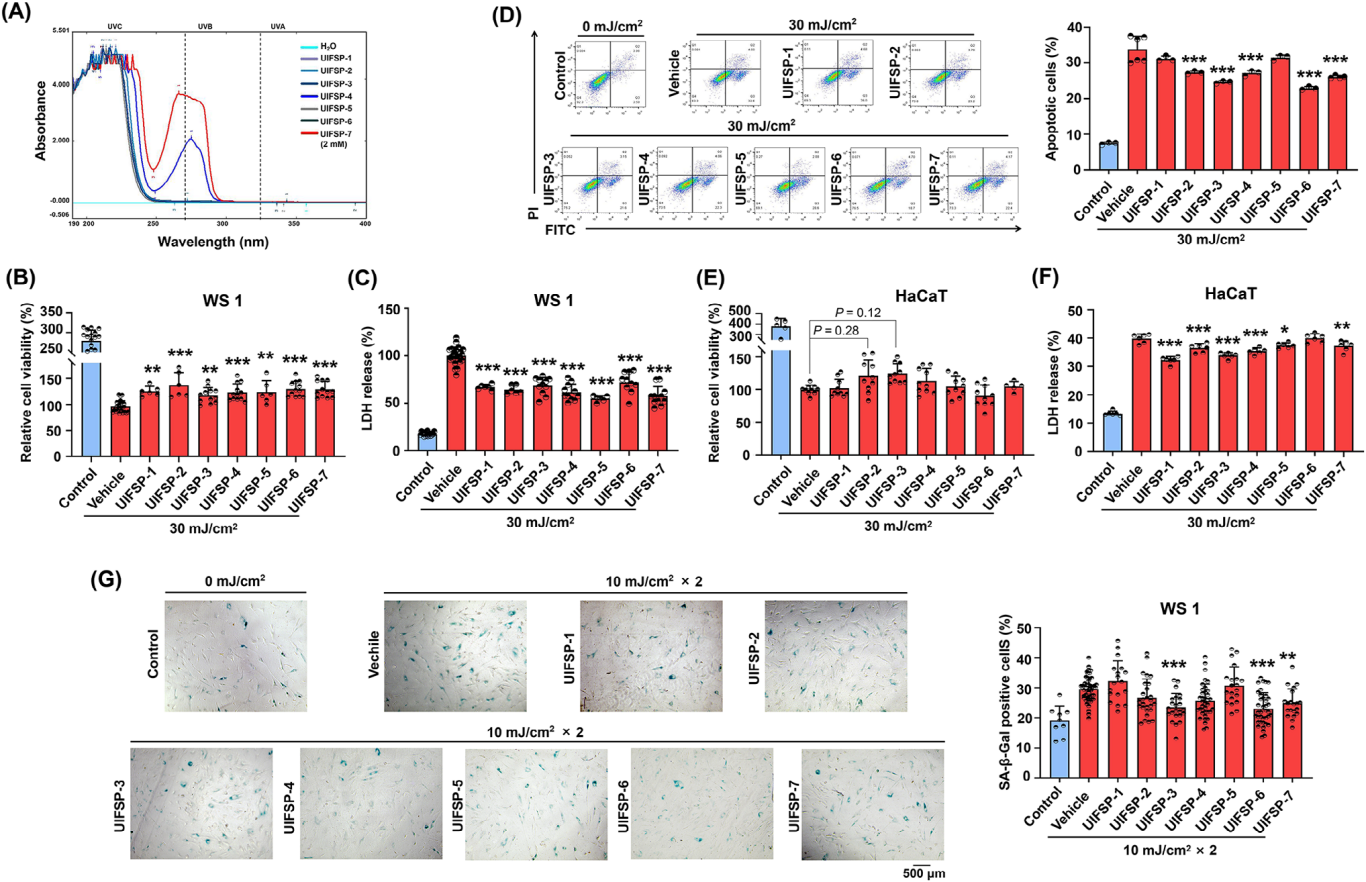
UV‐induced frog skin peptides (UIFSPs) reduce ultraviolet B (UVB)‐induced death and senescence in skin cells. (A) Absorption spectrum in the ultraviolet region of UIFSPs solution. UIFSPs were dissolved in deionized water (2 mM) to measure the absorption spectra between 190 and 400 nm. The effect of UIFSPs on (B) cell viability, (C) lactate dehydrogenase (LDH) activities, (D) cell apoptosis, and (G) senescence in WS1 cells, as detected by Cell Counting Kit‐8 (CCK‐8)‐based assay, LDH release assay, AV/PI staining, and SA‐β‐Gal staining analysis, *n *= 5. The effect of UIFSPs on (E) cell viability and (F) LDH activities of HaCaT cells measured by CCK‐8‐based assay and LDH release assay, *n *= 5. Skin cells were pretreated with UIFSPs (20 µM) for 48 h, exposed to 30 mJ/cm^2^ UVB once or 10 mJ/cm^2^ UVB twice, then detected cell death 36 h (WS1) or 24 h (HaCaT) later and senescence 72 h later. Student's *t* test and one‐way analysis of variance (ANOVA) were used to evaluate the differences between groups. *p < 0.05, **p < 0.01, and ***p < 0.001, compared to the vehicle group.

We first explored the potential of natural UIFSPs as sun blockers with UV absorption abilities. The absorbance of the UIFSP samples were measured using a UV‐visible spectrophotometer, which revealed distinct absorption peaks within the 190‐400 nm range for the polypeptide (Figure 3A). Specifically, the absorbance associated with peptide bonds was primarily observed between 190‐250 nm. Notably, peptides containing aromatic amino acid residues (tyrosine [Tyr, Y] within the UIFSP sequences) showed absorption in the 250−300 nm (UVB 280−320 nm), with a maximum peaks at 280 nm. Therefore, UIFSP‐4 (GGYDVDKNNSRLK), which contains one tyrosine residue, and UIFSP‐7 (SGGYGGGSRDYYS), which contains three tyrosine residues, had direct absorption capacities (Figure 3A).

To further verify the biological effects of UIFSP, UIFSP‐treated skin cells were exposed to UVB irradiation (30 or 10 mJ/cm^2^). Under a dose of 30 mJ/cm^2^, UVB exposure caused prominent toxic effects on WS1 cells, whereas UIFSPs increased cell viability and mitigated cell death with cell membrane integrity (Figure 3B,C). Exposed cells at 30 mJ/cm^2^ also exhibited irreversible hallmarks of both apoptosis and necrosis, while UIFSP‐6 decreased cell apoptosis by 32.29% particularly (Figure 3D). In addition, we explored the potential of UIFSPs on cell senescence after a lower dose UVB exposure (10 mJ/cm^2^) because UV radiation is the most influential extrinsic factor in skin aging. As shown in Figure 3G, a significant decrease in SA‐β‐Gal activity by 21.01% was observed in UVB‐exposed cells with UIFSP‐6 treatment. Besides, we also performed cell viability and lactate dehydrogenase (LDH) release assays in human keratinocytes (HaCaT cells), the outer layer of skin which showed more prominent toxicity than fibroblasts 24 h post UVB radiation. UIFSPs advanced slightly HaCaT cell vigor but reduced cell cytotoxicity significantly (Figure 3E,F). We noticed that each UIFSP exerted different positive effects, which may contribute to the resistance of frog skin to UVB exposure.

### 2.4 PTD‐coupled UIFSP fusion polypeptides enhance the photoprotective effects of UIFSPs

**FIGURE 4:**
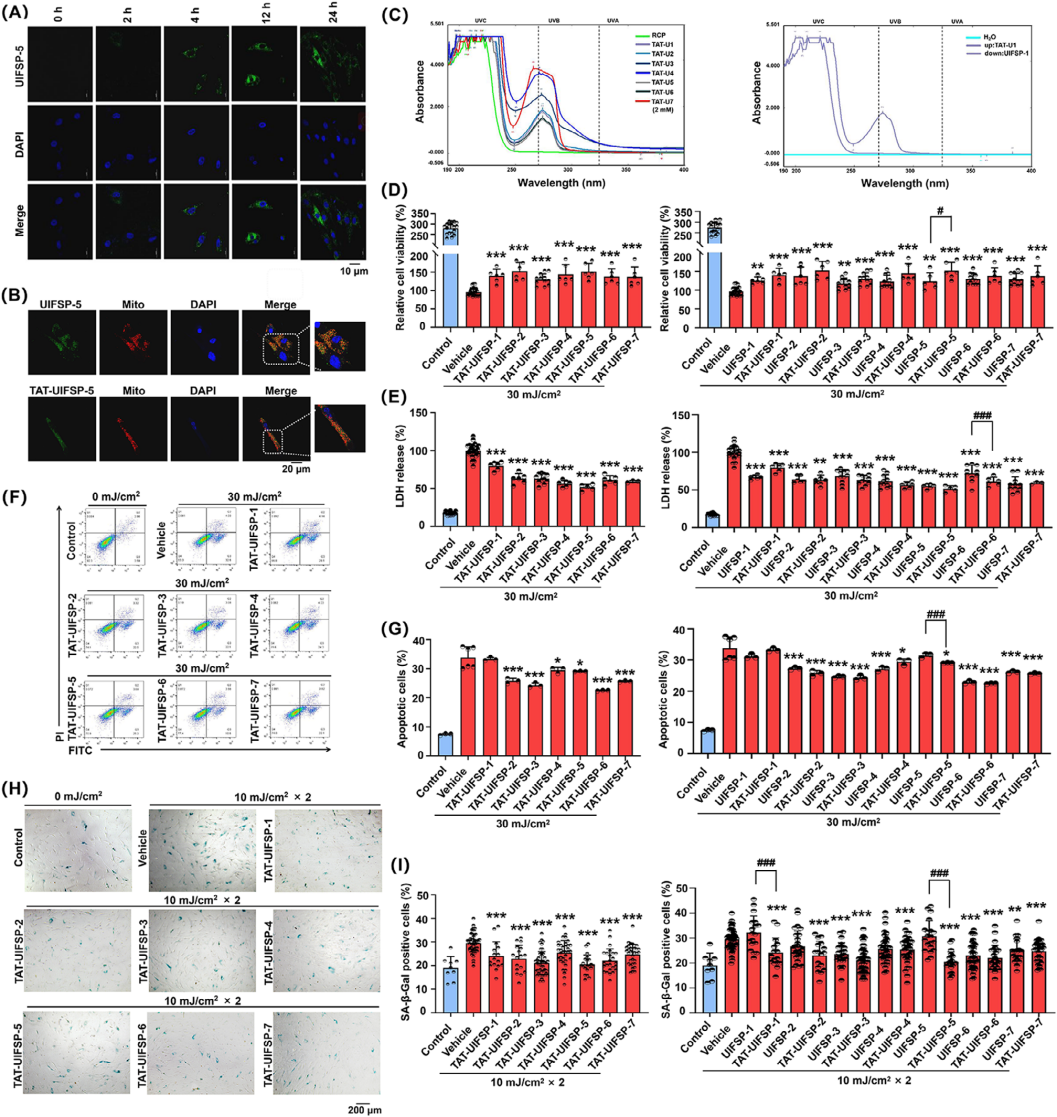
Transcription trans‐activating (TAT)‐conjugated UV‐induced frog skin peptides (UIFSPs) increase the efficacy of UIFSPs in reducing ultraviolet B (UVB)‐induced skin damage. (A) Time‐dependent membrane impermeability of FITC‐labeled RIFSP‐5 to WS1 cells through confocal microscopy. WS1 cells were treated with FITC‐labeled UIFSP‐5 at a concentration of 20 µM for 2, 4, 12, and 24 h, respectively. Nuclei (blue) were stained with the DNA‐binding dye DAPI 4',6‐diamidino‐2‐phenylindole. Scale bars, 10 µm. (B) Confocal microscopic analysis for colocalization of UIFSP‐5/TAT‐conjugated UIFSP‐5 with mitochondrion. WS1 cells were treated with FITC‐labeled UIFSP‐5 or TAT‐conjugated UIFSP‐5 at a concentration of 20 µM for 24 h. Mitochondria (red) were stained with the Mito‐Tracker Red CMXRos. Scale bars, 20 µm. (C) Absorption spectrum in the ultraviolet region of TAT‐conjugated UIFSPs solution. TAT‐conjugated UIFSPs were dissolved in deionized water (2 mM) to measure the absorption spectra between 190 and 400 nm. Influence of TAT‐conjugated UIFSPs on (D) viability, (E) LDH activities, (F) and (G) apoptosis rate, and (H) and (I) senescence of WS1 cells, as detected by Cell Counting Kit‐8 (CCK‐8)‐based assay, LDH release assay, AV/PI staining, and SA‐β‐Gal staining analysis, respectively, *n *= 5. WS1 cells were pretreated with TAT‐conjugated UIFSPs (TAT‐UIFSPs) (20 µM) for 48 h, exposed to 30 mJ/cm^2^ UVB once or 10 mJ/cm^2^ UVB twice, and detected 36 h (WS1), 24 h (HaCaT), or 72 h (WS1) later. Student's *t*‐test and one‐way analysis of variance (ANOVA) were used to evaluate the differences between groups. *p < 0.05, **p < 0.01, and ***p < 0.001, compared to the vehicle group. *^#^p* < 0.05, *^###^p *< 0.001, statistical difference between UIFSP and TAT‐conjugated UIFSP group.

Peptide drug candidates were reported against a range of molecular targets, such as historically dominant extracellular hormone receptors or intracellular targets. Therefore, we used FITC‐labeled UIFSP‐5 to evaluate its possible intracellular uptake by skin cells. As shown in Figure 4A, weak green fluorescence could be observed in WS1 cells 2 h after administration of fluorescein isothiocyanate (FITC)‐labeled UIFSP‐5, and an obvious intracellular FITC signal after administration for 24 h. To increase intracellular peptide concentration, we fused a protein transduction domain (PTD) to the UIFSPs because the transcription trans‐activating (TAT) sequence (YGRKKRRQRRR) as the first PTD^19^ helps its conjugated cargo to penetrate cellular membranes efficiently (Figure S5D). Moreover, through subcellular localization analysis, we detected obvious co‐localization of FITC‐labeled UIFSP‐5 or TAT‐conjugated UIFSP‐5 with the MitoTracker signal (Figure 4B). All of the TAT‐UIFSPs absorbed the UVB light due to the existence of tyrosine residues in TAT sequences (Figure 4C and Figure S5C). In WS1 cells, modification with TAT sequences significantly enhanced the protective abilities of UIFSPs. For example, the viability of UIFSP‐5 group increased from 123% to 151% in TAT‐conjugated UIFSP‐5 group (Figure 4D). Similarly, in reducing cell death and apoptosis, the beneficial effects of the less potent peptides were significantly amplified,  including a reduction in LDH release from 72% in the UIFSP‐6 group to 61% in the TAT‐conjugated UIFSP‐6 group (Figure 4E), as well as the apoptosis rate from 31% in the UIFSP‐5 group to 29% in the TAT‐conjugated UIFSP‐5 group (Figure 4F,G). In addition, UIFSP‐5 did not reduce SA‐β‐Gal activity while TAT‐conjugated UIFSP‐5 decreased by 31.68% after UVB radiation (Figure 4H,I). These results demonstrated the effectiveness of UIFSPs with TAT modification to strengthen UIFSP function, especially the UIFSPs with less membrane penetration potency.

### 2.5 UIFSPs prevent and repair UVB‐induced skin injury in vivo

**FIGURE 5:**
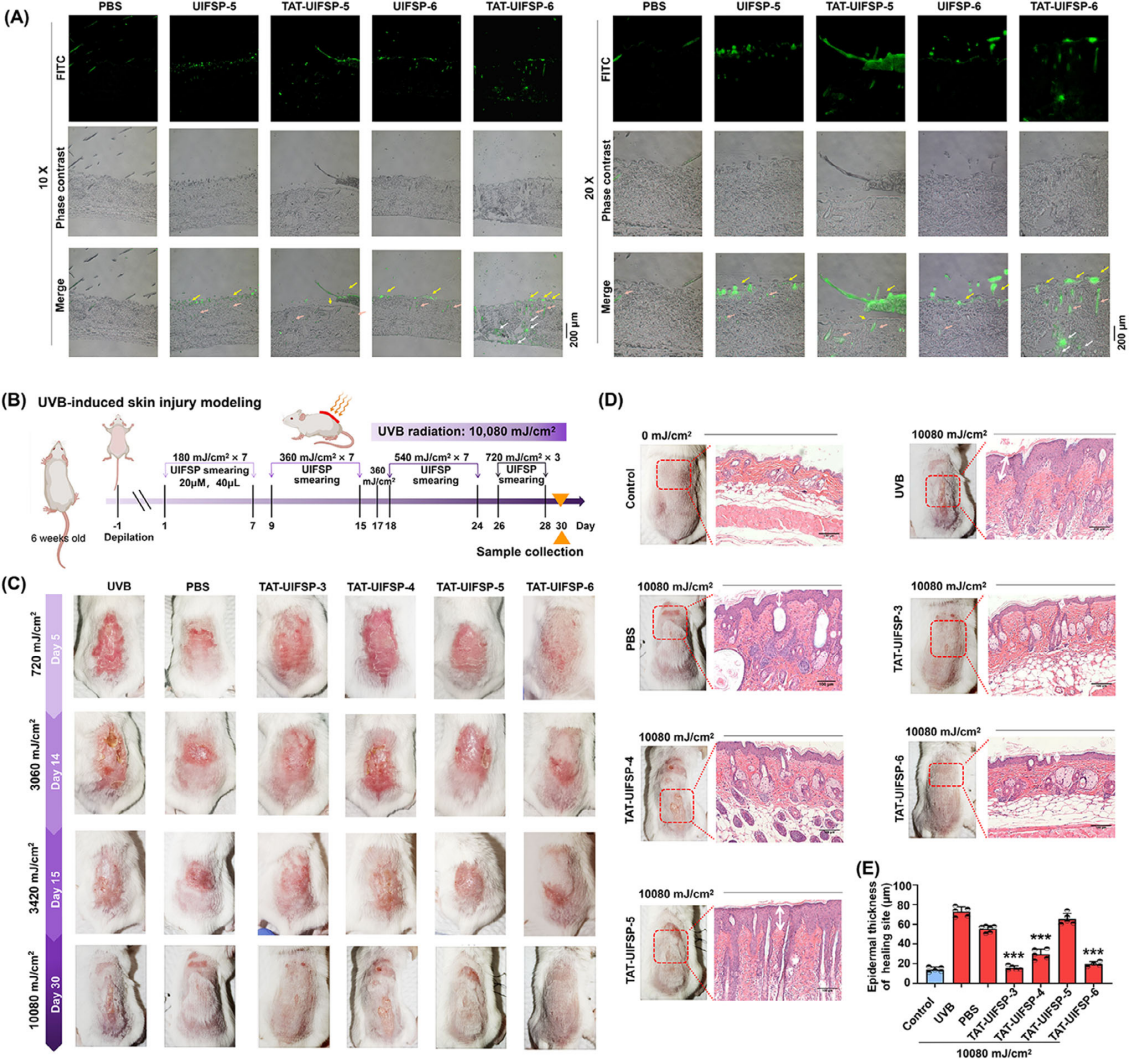
UV‐induced frog skin peptides (UIFSPs) attenuate skin photodamage and promote wound healing in mice. (A) Penetration of UIFSPs or transcription trans‐activating (TAT)‐conjugated UIFSPs into rat skin. Frozen sections of skin tissues were obtained 24 h after the application of indicated peptides (20 µM, 100 µL) and observed by a fluorescent microscope. Scale bars, 200 µm. (B) Schematic diagram of the time course of ultraviolet B (UVB)‐induced skin injury modeling. The dorsal skin of mice was irradiated with UVB light for 30 days. During this period, 20 µM TAT‐conjugated UIFSP‐3, ‐4, ‐5, ‐6 (TAT‐UIFSP‐3, ‐4, ‐5, ‐6) or PBS was smeared on the irradiated area after UVB radiation. The dorsal skin tissues were collected at the end of the experiment. (C) Macroscopic changes in the dorsal skin on Day 5, 14, 15, and 30 during UVB exposure from each group. (D and E) Histology and epidermal thickness quantification of the mice skin with or without TAT‐conjugated UIFSPs (TAT‐UIFSPs) treatment 24 h after UVB exposure (10,080 mJ/cm^2^). Skin sections were stained with Hematoxylin‐eosin (H&E), scale bars, 10 µm, *n *= 5. Student's *t‐*test or one‐way analysis of variance (ANOVA) was used to evaluate the differences between groups. **p < 0.01 and ***p < 0.001, compared to the UVB group.

Before verification of UIFSPs function in vivo, we determined their capability to promote cell migration in vitro wound healing (Figure S4). Next, we established a cutaneous photodamage model in mice through daily exposure to UVB radiation.The initial exposure dose was set at 180 mJ/cm^2^ and the dosage was gradually increased to a maximum of 720 mJ/cm^2^ by the final exposure. The irradiation field on back skin was topically spread PBS or TAT‐conjugated UIFSP‐3, ‐4, ‐5, ‐6 (Figure 5A). As shown in Figure 5B, the mice exposed to 720 mJ/cm^2^ showed visible physical changes as the formation of erythema at Day 4, and cutaneous wounds aggravated and reached a maximum at Day 14 (3060 mJ/cm^2^) but translated into injury repair at Day 15 (3420 mJ/cm^2^). Throughout this period, skin sprayed with TAT‐conjugated UIFSP‐3 incurred less severe damage than control UVB group. At Day 30 (10,080 mJ/cm^2^), two areas of green wounds existed in the phosphate buffered saline (PBS) groups, and poor wound healing with obvious scars existed in the vehicle groups. In contrast, mice in the TAT‐conjugated UIFSP‐3 and UIFSP‐6 groups experienced more rapid wound healing with minimal or no scarring (Figure 5B). H&E images of pathological scarring characterized thick epidermis in the vehicle group, but TAT‐conjugated UIFSP‐3 and 6 displayed a thinner epidermis close to normal state (Figure 5C,D). Together, the above data demonstrated that UVB‐induced frog skin peptides protected skin from UVB radiation.

We also investigated the ability of FITC‐labeled TAT‐conjugated UIFSP‐5 or ‐6 to penetrate the mouse skin. Given the short half‐life of peptides, frozen sections of skin tissues were obtained 24 h after the application of FITC‐labeled TAT‐conjugated UIFSP‐5 or ‐6. Observation by fluorescent microscope displayed that there was no specific fluorescence in PBS‐treated mouse skin and a weak fluorescence overlying the surface of epidermis and in hair follicle of mouse skin within UIFSP‐6 or ‐5 or TAT‐UIFSP‐5 treatment (Figure 5E). TAT‐conjugated UIFSP‐6 was able to penetrate the normal epidermal barrier, and most of peptides are located under the epidermis, especially at the junction of epidermis and dermis, thereby giving play to their photoprotective roles. In terms of potential toxicity, the UIFSPs and TAT‐conjugated UIFSPs were predicted to be nontoxic peptides through ToxinPred software (https://webs.iiitd.edu.in/raghava/toxinpred/index.html). Moreover, to ensure the safety of UIFSPs in vivo, we also established two kinds of mouse models and administered UIFSPs and TAT‐conjugated UIFSPs through external use or subcutaneous injection. No visible abnormalities were observed, and a histological examination revealed no significant differences among skin tissues treated with UIFSPs and TAT‐conjugated UIFSPs (data not shown).

### 2.6 UIFSPs provide photoprotection on skin cells via complex mechanisms

**FIGURE 6:**
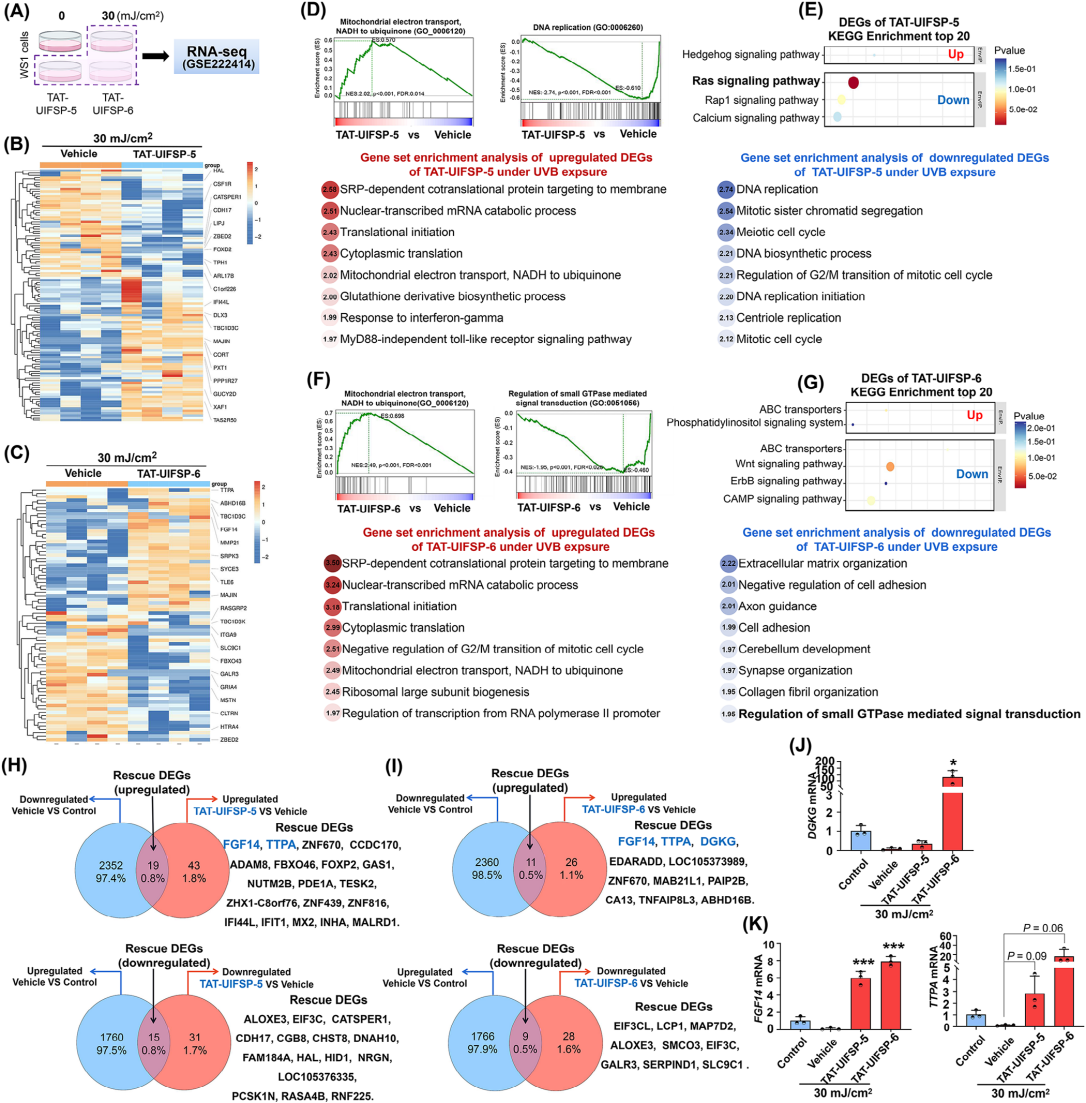
The landscape of genes affected by transcription trans‐activating (TAT)‐conjugated UV‐induced frog skin peptides (UIFSPs) after ultraviolet B (UVB) exposure. (A) Global RNA‐seq assumption of WS1 cells with TAT‐conjugated UIFSP‐5 or ‐6 (TAT‐UIFSP‐5 or ‐6) treatment, mRNA extracted 24 h after 30 mJ/cm^2^ UVB. (B and C) Heatmap of the global differentially expressed genes between TAT‐conjugated UIFSP‐5 or ‐6 (TAT‐UIFSP‐5 or ‐6) group with vehicle group. (D and F) Gene set enrichment analysis (GSEA) showed representative Gene Ontology (GO) terms and pathways in the TAT‐conjugated UIFSP‐5 or ‐6 (TAT‐UIFSP‐5 or ‐6) group. The color keys from white to red or blue indicate the enrichment levels (Normalized Enrichment Score [NES]) from low to high. (E and G) Kyoto Encyclopedia of Genes and Genomes (KEGG) pathway analysis of differentially expressed genes (DEGs) in the TAT‐conjugated UIFSP‐5 and ‐6 (TAT‐UIFSP‐5 and ‐6) group. (H and I) Venn diagram showing the “rescue DEGs,” which was down/upregulated by UVB but up/downregulated by TAT‐conjugated UIFSP‐5 and ‐6 (TAT‐UIFSP‐5 and ‐6). (J and K) qRT‐PCR analysis of the preferred rescue DEGs in TAT‐conjugated UIFSP‐6 (TAT‐UIFSP‐6) group and the overlapped rescue DEGs between TAT‐conjugated UIFSP‐5 and ‐6 (TAT‐UIFSP‐5 and ‐6) group for the validation of RNA‐Seq data, *n *= 3.

To analyze the underlying mechanism of UIFSPs against UVB exposure, we profiled gene expression as shown in Figure 6A. Note that 36‐h post exposure was used to study the influence of UIFSPs on fibroblast and 24‐h post exposure was used to probe their regulation mechanism. Preferentially differentially expressed genes were identified, and the data are accessible at the GEO database (accession No. GSE222414). Figure 6B,C shows the heatmap of changed genes upon TAT‐conjugated UIFSP‐5 or ‐6 treatments, implying the changed transcriptional profile of WS1 cells after UVB exposure. Functional enrichment analysis extracts the underlying biological interpretation shown in Figure 6D,F. For example, peptide‐downregulated genes were mostly related to DNA replication and cell cycle, suggesting that UIFSP could prevent compromised cells from entering next cell cycle after UVB radiation. In addition, protein translation and mitochondrial electron transport were upregulated in both two peptide groups, revealing an enhancement of energy production and biosynthesis by UIFSP in response to UVB radiation. Interestingly, there were also similar functional enrichment results of down‐regulated genes between TAT‐conjugated UIFSP‐5 and ‐6 groups, as for TAT‐conjugated UIFSP‐5‐inhibited Ras signaling pathway (Figure 6E) while TAT‐conjugated UIFSP‐6 negatively regulated small GTPase‐mediated signal transduction (Figure 6G).

In our analysis of differential gene expression, we identified a subset of genes termed “rescue DEGs” (Figure 6H,I)which exhibited a reversal in expression patterns following TAT‐UIFSP treatment in comparison to the vehiclel group at 30 mJ/cm². Specifically, “upregulated rescue DEGs” refer to overlapped genes that were downregulated by UVB compared with control group (0 mJ/cm²) but upregulated by TAT‐UIFSP treatment compared with UVB gruop (30 mJ/cm²). Conversely, “downregulated rescue DEGs” are genes that were upregulated by UVB and subsequently downregulated by TAT‐UIFSP treatment. The validation results of three rescue DEGs from qRT‐PCR were consistent with RNA‐Seq. In Figure 6J, the mRNA levels of *FGF14* and *TTPA* were both upregulated in the TAT‐conjugated UIFSP‐5 or ‐6 group, and *DGKG* was upregulated in the TAT‐conjugated UIFSP‐6 group (Figure 6K). *FGF14* was previously reported to inactivate the mitogen‐activated protein kinase (MAPK) signaling pathway,^20^ downstream of small G‐proteins.^21^ And in Figure S7A,D, the Gene Ontology (GO) term of MAPK binding wass upregulated in TAT‐conjugated UIFSP‐5 group and phosphatidylinositol‐3‐phosphatase activity was downregulated in TAT‐conjugated UIFSP‐6 group. Therefore, we inferred that small GTPase or RAS‐mediated signal transduction and the MAPK and PI3K/AKT signaling can be attributed to one of the efficiency mechanisms of UIFSPs.

### 2.7 UIFSPs suppress AKT and MAPKs activation after UVB radiation

**FIGURE 7:**
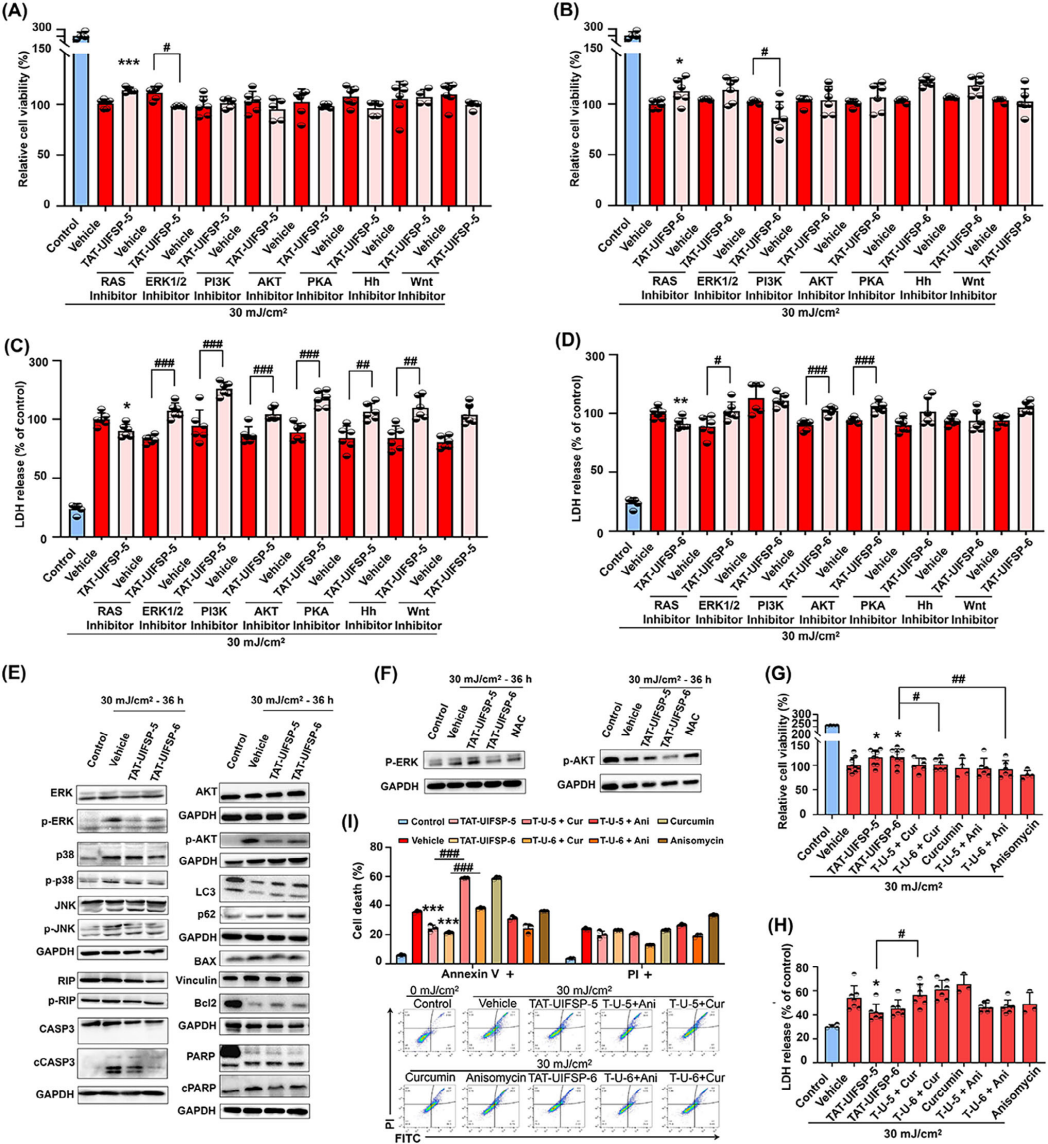
Inactivation of Ras/PI3K/AKT and Ras/MAPKs pathway are involved in the photoprotection of UV‐induced frog skin peptides (UIFSPs). Influence of various inhibitors of designated signaling on (A) and (B) cell viability, and (C) and (D) LDH activities of irradiated skin cells pretreated with TAT‐conjugated UIFSP‐5 or ‐6 (20 µM), *n *= 6. The concentration of the inhibitors: RAS inhibitor Abd‐7 (50 µM), ERK1/2 inhibitor 1 (10 µM), PI3K‐IN‐30 (5 µM), AKT‐IN‐1 (10 µM), PKA inhibitor (6‐22) amide TFA (10 µM), Hedgehog (Hh) inhibitor cyclopamine (5 µM), and Wnt/β‐catenin inhibitor MSAB (5 µM). (E and F) Western blotting showing the change in the expression of protein markers related to the Ras‐PI3K‐AKT signaling and MAPK pathways and cell death. Western blotting analysis was performed for at least three independent experiments. WS1 cells were pretreated with TAT‐conjugated UIFSP‐5 or ‐6 (TAT‐UIFSP‐5 or ‐6) and exposed to ultraviolet B (UVB) (30 mJ/cm^2^) and detected 36 h later. (G–I) Viability, LDH activities, and apoptosis rate of WS1 cells 36 h after UVB exposure, *n *= 6. Cells were pretreated with TAT‐conjugated UIFSP‐5 or ‐6 (TAT‐UIFSP‐5 or ‐6) (20 µM), anisomycin (1 µM) or curcumin (20 µM), TAT‐conjugated UIFSPs plus anisomycin (T‐U + ani), or TAT‐conjugated UIFSPs plus curcumin (T‐U + cur). Student's *t* test and one‐way analysis of variance (ANOVA) were used to evaluate the differences between groups. *p < 0.05 and ***p < 0.001, compared to the vehicle group. *^#^p* < 0.05, *^##^p *< 0.01, *^###^p *< 0.001, statistical difference between two specified groups.

To validate the pathways shown in the enrichment analysis, we introduced inhibitors targeting RAS‐ERK, PI3K‐AKT, cAMP‐PKA, Wnt/β‐catenin, and Hedgehog (Hh) signaling. Results from Cell Counting Kit‐8 (CCK‐8, Beyotime) analysis revealed that administration of RAS‐ERK and PI3K‐AKT inhibitor blocked the promotion of cell proliferation by UIFSP‐5/6 against UVB, whereas inhibitor of PKA and Hh exhibited limited effect in cells treated with UIFSP‐6 but not UIFSP‐5 in response to UVB (Figure 7A,B). Consistently, administration of the above inhibitors significantly increased the release of LDH for cells treated with UIFSP‐5/6 (Figure 7,D). In our study, UVB‐induced activation of AKT and MAPKs in fibroblasts, whereas TAT‐conjugated UIFSP‐5 and ‐6 inhibited phosphorylation of ERK, p38 MAPK, and JNK and AKT along with a decrease in cell death as necrosis and apoptosis but an increase of cell autophagy after UVB radiation (Figure 7E). Moreover, the antioxidant activity was not contributed to the inhibition of AKT and MAPK pathways (Figure 7F).

To investigate whether the protective peptides targeted MAPKs signaling, we further pretreated WS1 cells with TAT‐UIFSPs in combination with the ERK agonist curcumin^22^ and the JNK agonist anisomycin.^23^ Curcumin and anisomycin increased the phosphorylation of MAPK proteins in peptide‐treated WS1 cells after UVB exposure (Figure 7F). Consequently, curcumin and anisomycin aggravated UVB‐induced cell damage, for example, cell apoptosis, which was significantly reduced by TAT‐conjugated UIFSP‐6. While in comparison with TAT‐conjugated UIFSP‐5 and UIFSP‐6 groups, curcumin and anisomycin ablated the protective effects of TAT‐conjugated UIFSPs, showed by decreased viability and aggravated cytotoxicity and apoptosis after UVB exposure (Figure 7G–I). Therefore, these results indicated that suppressing MAPK activation contributes to the photoprotective effects of UIFSPs in skin cells after UVB exposure.

## 3 DISCUSSION

When the intensity of UV irradiation is more than 3.7 mJ/cm^2^ per day, the redox homeostasis of skin can be destroyed, which triggers the generation of oxidative stress, inflammation, immunosuppression, mitochondrial dysfunction, altered intracellular communication, genomic instability, apoptosis, and matrix‐metalloproteases production leading to the onset of photo‐carcinogenesis and photo‐aging.^11^, ^24^ At the molecular level, these changes occur via activation of several protein kinases as well as transcription pathways, formation of ROS, and release of cytokines together. Among the initial events, UV has a direct impact on the activation of epidermal growth factor receptor and various kinases (e.g., Ras and Rac, phosphatidylinositol 3‐kinase [PI3K], ribosomal S6 kinases [RSK], and ataxia telangiectasia mutated [ATM]) transfer external damaging signals to the nucleus.^25^

Photoprotective strategies aim to mitigate the detrimental effects of UV radiation through various mechanisms, such as the direct blockade of UV photons, DNA damage repair, neutralization of ROS, reduction of inflammation, and modulation of immune responses.^26^ However, there are still limited curative efficacy and some toxic effects in the photochemical derivatives acting as sunscreens or counteracting the effects of UV radiation. Intriguingly, amphibian skin, a keratinized tegument bringing the evolutionary water‐land gap, evolved several strategies to protect skin from both endogenous and exogenous injuries, representatively skin‐bearing glands with structural physiological functions and chemical defenses, especially those composed of gene‐encoded peptides/proteins.^27^, ^28^ Amphibian skin peptides play a major role in maintaining skin integrity and functionality, and each amphibian species produces its own specific set of peptides with well‐defined sequences and biological effects.^9^

Since therapeutic peptides become one of the hottest topics in pharmaceutical research by strengths of high potency, specificity, and good safety profile,^29^ focusing on the discovery of organisms‐derived biopeptides seems be able to provide additional and species‐specific drug resources. In our study, we used *P. nigromaculatus*, a dark‐spotted frog occupying a broad range of habitats. First, we explored the skin adaptability and resistance of *P. nigromaculatus* to the exogenous radiation exposure. Unlike typical electronic stimulation in previous studies,^10^ a serial dose of UVB was administered to the dark‐spotted frog as a stimulus. Specifically, one leg of frog was exposed to UVB (150 mJ/cm^2^) by four times in 1 week, and the other leg was shielded for the negative control. We observed that frog skins exhibited no obvious photodamage but rather an enhancement in gland activity.

Previous studies have found that amphibian skin constellated with a large variety of gene‐encoded peptides/proteins, serving as an evolved strategy to protect skin from both endogenous and exogenous insults. And the UV‐VIS and multi‐omics in our recent research, has uncovered significant alterations in the activity of peptidases or endopeptidases within irradiated skin tissues.^17^ In the current study, we examined the peptide profile after UVB radiation, in which skin tissues were grinded for sonication and the supernatants were applied to 10 kDa MWCO filters. The results showed an increased expression of small molecule gene‐coding peptides (named UIFSPs) after intermittent UVB exposure, and peptide annotation revealed that UVB may act on structural proteins and induce UIFSPs production.

Despite UIFSPs as skin products under a non‐injured UVB dose, whether they were cleavage products from endogenous proteins because of photon attack was still unknown. If the radiation has sufficient energy, a molecule absorbs EM radiation and its electron can escape the coulomb attraction of the nucleus, and the molecule is ionized, whereas non‐ionizing UVR exposure caused molecules to undergo rotational or vibrational transitions and experience minimal changes in the stability of the electron‐nucleus attraction, resulting in negligible chemical effects.^8^ It suggested that the production of UIFSPs might be a consequence of possible physiological action in UV‐irradiated frog skin. Among the seven significantly upregulated UIFSPs, only UIFSP‐6 is derived from extracellular matrix protein (collagen). While a great majority of secretory proteins carry a short peptide at their N‐terminals, called signal peptide (SP),^30^ we thus explored the potential cleavage sites through PROSPER and conducted the signal peptide prediction via SignalP 5.0 (Figure S2). However, SignalP‐5.0 had not identified SPs in the UIFSP‐6 precursor, collagen type VI alpha 3 chain (g.68918). But the UIFSP‐7 precursor, cold‐inducible RNA‐binding protein‐like which enriched at the cytoplasm or vegetal cortex, was predicted to have a sec signal peptide (Figure S2G). The cleavage sites were predicted between residues 17 and 18, and residues 20 and 21, which means its SP was located at its N‐terminals (1‐20) while the UIFSP‐7 mapped the c‐terminal region of its precursor protein (151–163). In consequence, UIFSPs could not be cleavage products of the signal peptide from the preproteins for protein secretion. To further analyze the role of UIFSPs production from UV‐exposed frog skin, seven significantly upregulated peptides were synthesized to investigate their bioactivities.

Although eight antimicrobial peptides were identified from *P. nigromaculatus*,^16^ UIFSPs had no potency against pathogens (shown in Figure S2 and Table S5). In light of the secreted skin peptides with predominantly antioxidant activities,^31^ we detected the antioxidant ability of UIFSPs. Compared with the direct free radical‐scavenging activity of UIFSP‐4 and ‐7, UIFSPs were more capable of scavenging UVB‐induced ROS, and UIFSP‐6 significantly enhanced the endogenous antioxidase system in UVB‐exposed skin cells (Figure 2). It is reasonable to infer that UIFSPs could regulate UV‐induced oxidative stress in skin cells. We hypothesized that these UVB‐induced frog skin peptides may be superior in launching biological cascades that fuel their photoprotective abilities in skin cells. Therefore, cellular and animal level experiments were conducted to verify the effects of UIFSPs.

UV rays destroy redox homeostasis, form unstable ROS to damage biological molecules,^32^, ^33^, ^34^ and result in disruption of lipids function and structure via lipid peroxidation to develop compromised cell membranes.^35^ Therefore, exposure to UVB caused severe cell death in human keratinocytes and fibroblasts at a dose of 30 mJ/cm^2^. In contrast, UIFSPs reduced acute photodamage with potency including advance in cell viability, reduction in LDH release, and decrease in apoptosis percentage (Figure 3B–F). Moreover, skin photoaging is caused by chronic exposure to UV and accounts for more than 80% of facial aging.^36^ Our study illustrated the anti‐photoaging property of UIFSPs in fibroblasts exposed to a lower dose of 10 mJ/cm^2^ (Figure 3G). Amphibian skin can quickly repair itself after being damaged,^37^, ^38^ and UIFSPs in the present study also showed powerful wound‐healing activity (Figure S4).

Peptide drugs have been proven to disrupt protein–protein interactions,^39^ target extracellular hormone receptors,^40^ and inhibit intracellular targets.^41^ Here, we found the ability of UIFSPs to cross cell membranes and the colocalization of FITC‐peptide and MitoTracker in fibroblasts. Moreover, we modified UIFSPs with TAT sequence conjugation to facilitate the internalization of such heterogeneous peptides into cells,^42^ which enhanced therapeutic effects of UIFSPs against skin photodamage (Figure 4D–I). Our previous study demonstrated that with the insertion of membrane permeability elements, TAT‐RP1 penetrated the skin of rats and ameliorated radiation‐induced skin damage.^43^ In the present study, topical application of TAT‐conjugated UIFSP‐6 penetrated into mouse skin and played a therapeutic role in wound repair and normalizing skin structure after UVB exposure (10,080 mJ/cm^2^) (Figure 5B–E).

Studies have reported that organic sunscreens are composed of UV‐absorbing molecules with broad‐spectrum UV protection properties, such as natural antioxidants and polyphenols with conjugated π system which block UV light by absorbing it. To determine whether the protective effects of UIFSPs against UVB radiation were due to the presence of peptide to absorb UVB light in cells, we measured the absorbance of UIFSP samples using a UV‐visible spectrophotometer. According to the amino acid sequence and chemical structure of polypeptides, the UV absorption of proteins in the range of 190–250 nm is due almost entirely to π → π∗ transitions in the peptide bonds, and absorption in the range of 250−300 nm is dominated by the aromatic side‐chains of tyrosine (Tyr) in UIFSPs and TAT‐conjugated UIFSPs. As shown in Figure S5C, TAT sequences exhibited dose‐dependent UV‐absorbing capacity. Therefore, peptides including UIFSP‐4 (GGYDVDKNNSRLK), UIFSP‐7 (SGGYGGGSRDYYS), TAT (YGRKKRRQRRR), and all TAT‐conjugated UIFSPs could absorb the UVB light with absorption peaks at about 270−280 nm (UVB, 280−320 nm) (Figures 3A and 4C).

However, the functional evaluation of the control peptides via series of cellular and animal studies indicated that no matter a random combination of amino acid residues (randomized controlled peptides [RCP]) or TAT sequences had no physiological protective effects against UVB (Figures S5 and S6), which results showed that RCP increased UVB‐induced ROS level and apoptosis rate faintly in vitro (Figure S5), and in vivo, TAT‐conjugated RCP further aggravated UVB‐induced skin injuries in spite of the existence of tyrosine residue for UVB absorption (Figure S6). These studies hinted that the specific amino acid sequences of UIFSPs and their high bioactivity targeting endogenous physiological elements may contribute to the UV photo‐protection.

For better application of UIFSPs, we analyzed how UIFSPs regulate the skin cell response to UVB exposure by profiling gene expression in fibroblasts with TAT‐conjugated UIFSP‐5 or ‐6 treatment. Congruent changes were observed between two groups, including an increase in biosynthesis and mitochondrial electron transport, along with a significant decline in DNA replication and cell cycle regulation (Figure 6D–F and Figure S7). In Figure S8, UIFSPs reduced mitochondrial dysfunction after UVB exposure, which relieved oxidative stress in mitochondria, maintained mitochondrial membrane potential (△Ψm), and promoted ATP production. This allowed us to consider these UV‐induced peptides inspired skin cells with biosynthesis and energy production to counteract photon energy, meanwhile they redeployed cell cycle stage to block progression of photodamage. Of particular interest were the peptide‐regulated genes highlighted in Figure 6G,I, termed “rescue DEGs (upregulated)” which are genes whose UVB‐induced expression changes (down‐regulated) were reversed by UIFSPs (up‐regulated). For example, *DGKG* mRNA level was upregulated by TAT‐conjugated UIFSP‐6 (Figure 6J). DGKγ was reported to be an upstream suppressor of Rac1, which suppressed NADPH oxidase activation and ROS overproduction.^44^, ^45^ TAT‐conjugated UIFSP‐5 and ‐6 both upregulated *FGF14* and *TTPA* (Figure 6K). It was found that *TTPA* encoded α‐tocopherol transfer protein (α‐TTP) to transport α‐tocopherol (α‐Toc), a form of vitamin E with protection against radiation‐induced gastrointestinal injury.^46^ *FGF14* has been reported to inhibit the phosphorylation of p38, extracellular signal‐regulated kinase 1/2, and c‐Jun N‐terminal kinase.^20^, ^47^

Altogether, TAT‐UIFSP‐5 downregulated Ras signaling pathway (Figure 6E) and reregulated MAPK activities (Figure S7A), and TAT‐UIFSP‐6 downregulated small GTPase‐mediated signal transduction (Figure 6F) and phosphatidylinositol‐3‐phosphatase activity (Figure S7D), as well as in case of FGF14 (Figure 6K), the overlapped “up‐regulated rescue genes” has been reported to inhibit the phosphorylation of MAPKs. Therefore, we validated the RAS signaling and primarily MAPK pathways (Figure 7A–D), in which UVB exposure was reported to activate AKT and MAPK signaling pathways.^48^, ^49^ Our results demonstrated that UIFSPs suppressed the phosphorylation of MAPK‐related proteins and AKT, along with the inhibition of death‐associated protein expression after UVB exposure (Figure 7E). However, cell autophagy was induced by UIFSPs in UVB‐exposed fibroblasts (Figure 7E). The role of PI3K/AKT/mTOR and MAPK signaling^50^ in the modulation of autophagy has been widely accepted and studies reported protection of epidermal cells against UVB‐induced apoptosis through activating autophagy.^51^ In this study, UIFSPs stimulated autophagy and inhibited apoptosis in UVB‐exposed fibroblast, as shown in Figure S9E,F. Furthermore, the protective effects of UIFSP were ablated via addition of MAPK agonists, indicating that suppressing MAPK signaling pathway may be responsible for UIFSP protection.

In conclusion, this study illustrated that the skin of dark‐spotted frog could serve as a source of biologically active and functional molecules. By applying UVB radiation to frog skin, we identified a series of frog skin peptides (UIFSPs) with photoprotective effects and further modified UIFSPs with TAT sequence conjugation to enhance antiphotodamage efficacy in vitro and in vivo. Mechanistically, these UIFSPs negatively regulated Ras/AKT and Ras/MAPK signaling to prevent skin from UVB damage. Our studies demonstrated the possibility that inflicting specific stimuli on creatures with fascinating vitality and adaptation is an unappreciated strategy to screen functional biomolecules and develop potential therapeutic agents.

## 4 MATERIALS AND METHODS

### 4.1 Frog radiation inducibility

Artificially bred and farmed adult dark‐spotted frogs (*P. nigromaculatus*) were obtained from a breeding base in Sichuan Province, China. Before the experiments commenced, frogs were housed collectively in a 50 cm × 60 cm container with mealworms provided ad libitum for 7 days of acclimation. To establish a local skin irradiation model, frogs were exposed to a spectral peak at 312 nm of UVB radiation (BIO‐SUN, Vilber) at an intensity of 2.7 mW/cm^2^ and a dose of 150 mJ/cm^2^ per day, and the next radiation was performed with an interval of 1 day for a total of four exposures. During exposure, only the right frog leg was exposed to UVB, and the rest of the frog was shielded by foil. The frog experiment protocols were approved by the Animal Experimentation Ethics Committee of Sichuan University (Chengdu, China).

### 4.2 Peptidomic analysis

Skin tissues were mixed with four volumes of urea buffer (8 M urea, 1% protease inhibitor cocktail, 2 mM ethylenediaminetetraacetic acid (EDTA) for supersonic schizolysis, and the supernatants containing peptides were applied to 10 kDa MWCO filters. All peptide preparations were desalted with Ziptip C18 (ZTC18S960, Merck Millipore) for nano‐LC‐NSI‐MS/MS analysis. Peptidomic data analysis was partially conducted using PTM Bio.^52^ The detailed methods are described in the Supporting Information Materials and Methods.

### 4.3 Absorption spectra in the ultraviolet region

The UV absorbance of the peptide samples was measured using a UV–visible spectrophotometer (UV‐2550, Shimadzu). For UVB measurements, 350 µL of sample was loaded into a quartz cuvette and spectral acquisition (wavelength scanning) in the wavelength range of 190−400 nm was performed to record peptide absorbance in UV spectra.

### 4.4 Cell culture and UVB radiation

The WS1 cell line (human fibroblasts) was obtained from the American Type Culture Collection (ATCC), and the HaCaT cell line (human keratinocytes) was obtained from the German Cancer Research Center (Heidelberg, Germany).^53^ Cells were maintained in Dulbecco's modified Eagle medium (DMEM) supplemented with 1% antibiotics and 10% fetal bovine serum (BI) at 37°C in a humidified incubator with 5% CO_2_. Before UVB radiation, cells were pretreated with UIFSPs or TAT‐conjugated UIFSPs for 48 h. Upon reaching 90% confluence, cells were exposed to various intensities of UVB (10 or 30 mJ/cm^2^) under a thin layer of PBS. UVB radiation was conducted using a Vacuum UV aging test chamber (UVB‐15 W‐201) with an emission peak at 312 nm, and the strength of UVB was at an intensity of 2.7 mW/cm^2^.

### 4.5 Antioxidant activity assay

ABTS assay (Beyotime) was used to determine the free radical scavenging activity of UIFSPs solution or total antioxidant capacity (TAC) for UIFSP‐treated skin cells. Briefly, 10 µL of sample, including peptide solution and UIFSPs‐treated cell lysates, was mixed with 200 µL of diluted ABTS working solution at room temperature in the dark for 30 min. A decrease in absorbance at 415 nm indicated antioxidant activity. The rate of ABTS radical scavenging (%) was calculated by (*A*_blank_ − *A*_sample_) × 100/*A*_blank_, and the TAC was calculated by the prepared standard curve and normalized by protein concentration.

### 4.6 Cell viability assay

WS1 and HaCaT cells were seeded in a 96‐well plate at a density of 1 × 10^4^ cells per well.^17^ Then, cell viability was measured using the CCK‐8 according to the manufacturer's protocol, with a BioTek reader (Synergy HTX).

### 4.7 Cytotoxicity assay

UVB‐induced cell death was analyzed with the LDH cytotoxicity assay kit (Beyotime). To set the maximum enzyme activity group, lysate was added to WS1 and HaCaT cells 1 h before the experiment. Cell supernatant was obtained and relocated to a new 96‐well plate and then incubated with detection reagent, which was further detected with the BioTek reader (Synergy HTX). The rate of LDH release (%) was calculated by (*A*_sample_ − *A*_blank_)/(*A*_maximum_ − *A*_blank_) × 100.

### 4.8 Apoptosis assay

The annexin V‐FITC/PI apoptosis detection kit (Kaiji) was employed to analyze cell apoptosis. Cells were stained with annexin V (AV) and propidium iodide (PI) agent in working solution for 30 min and then tested by flow cytometry (BD FACSCelesta). Apoptotic cells were calculated by summing the percentage of early apoptotic cells, that is, annexin V (+) cells and PI (−) cells, and the percentage of late apoptotic annexin V (+) and PI (−) cells.

### 4.9 SA‐β‐galactosidase staining

To measure cellular senescence, skin cells were exposed to 10 mJ/cm^2^ UVB twice with an interval of 2 days. Then cells were fixed and followed by senescence‐associated β‐galactosidase (SA‐β‐gal) staining (Beyotime). More than 200 cells were counted in three randomized fields, and the percentage of blue‐stained senescent cells was counted using phase‐contrast microscopy (Olympus).

### 4.10 Mice skin photodamage modeling and treatment

Male BLAB/c mice from the same generation, 6 weeks old, were purchased from Dossy. The mice were randomly divided into the following groups (*n* = 6): control, model (UVB), negative control (UVB + PBS), and sample (UVB + TAT‐conjugated UIFSP‐3, ‐4, ‐5, and ‐6). Mice were shaved dorsal skin and then exposed to UVB radiation using a BIO‐SUN system with the UVB lamp at a peak emission of 312 nm (Vilber). Specifically, unrestrained mice were exposed to 180 mJ/cm^2^ within 7 consecutive days for the first week, 360 mJ/cm^2^ per day for the second week, 540 mJ/cm^2^ per day for the third week, and 720 mJ/cm^2^ per day for the last 3 days, for a total of almost 10 J/cm^2^. TAT‐UIFSP fusion peptides (20 µM) (dissolved in PBS) were administered immediately after exposure to irradiated skin sections.

### 4.11 Statistical analysis

The data are expressed as the mean ± standard deviation from at least three independent experiments. Unpaired two‐sided Student's *t* tests and one‐way analysis of variance followed by the post hoc Dunnett test were performed for difference analysis among samples using Prism 8 or SPSS software. Statistical significance was set at *p *< 0.05. Other methods are detailed in the Supporting Information Materials and Methods.

## AUTHOR CONTRIBUTIONS

S.Z. and T.Y. conceived and designed the study; T.Y., F.G., X.T., and Z.Y. carried out the molecular biology studies. T.Y., Y.L., B.S., and Z.T. performed the animal experiments. B.W., B.Y., and D.Y. provided guidance and advice. S.Z. and T.Y. analyzed the data and drafted the manuscript. All authors read and approved the final manuscript.

## CONFLICT OF INTEREST STATEMENT

The authors declare no conflicts of interest.

## ETHICS STATEMENT

All animal studies were approved by the Animal Experimentation Ethics Committee of Sichuan University (approval number: 2024092).

## Supporting information

Supporting Information

## Data Availability

The data of RNA‐seq are accessible through Gene Expression Omnibus series accession number GSE222414. The data that support the findings of this study are available from the corresponding author upon reasonable request.
