# Supplementary material for: UV radiation‐induced peptides in frog skin confer protection against cutaneous photodamage through suppressing MAPK signaling
Source: MedComm (2020). 2024 Jun 25;5(7):e625. doi: 10.1002/mco2.625 (PMC11196897; doi:10.1002/mco2.625)
Supplement: Supplementary file 1 — Supporting Information [file MCO2-5-e625-s001.docx]

**Title page**

**UV radiation-induced peptides in frog skin** **confer protection against**

**cutaneous photodamage through suppressing MAPK signaling**

Tingyi Yang^a^, Fenghao Geng^a^, Xiaoyou Tang^a,b^, Zuxiang Yu^a^, Yulan Liu^c^, Bin Song^a^, Zhihui Tang^a^, Baoning Wang^a^, Bengui Ye^b^, Daojiang Yu^c^, Shuyu Zhang^a,b,c,d,^*

^a^*Laboratory of Radiation Medicine,* *West China School of Basic Medical Sciences & Forensic Medicine,* *Sichuan University, Chengdu 610041, China.*

^b^*Medical College of Tibet University, Lasa 850002, China*

^c^*The* *Second Affiliated Hospital of Chengdu Medical College, China National Nuclear Corporation 416 Hospital, Chengdu 610051, China.*

*^d^NHC Key Laboratory of Nuclear Technology Medical Transformation (Mianyang Central Hospital), Mianyang 621099, China.*

**^*^****Corresponding author:**

**Zhang S**, Laboratory of Radiation Medicine, West China School of Basic Medical Sciences & Forensic Medicine, Sichuan University, Chengdu, 610041, China; E-mail address: [zhang.shuyu@hotmail.com](mailto:zhang.shuyu@hotmail.com) or zhangshuyu@scu.edu.cn.

**Running title:** Protective peptides from frog skins

**Supplementary Information**

1. **Materials**

**1.1** **Supplementary Table S1. The primers in qPCR**

| **Gene No.** | **Gene Name** | **Primer Type** | **Primer Sequence (5' to 3')** |
| --- | --- | --- | --- |
| **1** | **FGF14** | 1-Forward | CGGCAACCTGGTGGATATCTT |
|  |  | 1-Reverse | CCTTGAGCTGGGGATCTTGG |
| **2** | **TTPA** | 2-Forward | CCTCCTAAAGGCTGGCTACC |
|  |  | 2-Reverse | TCTTGGCTACGGATGGAGTG |
| **3** | **DGKG** | 3-Forward | GGATGACGTTTCACCGCAAA |
|  |  | 3-Reverse | GGGGTTGAGCAGATAGTGGAAT |
| **4** | **GAPDH** | 4-Forward | CCTTCCGTGTCCCCACT |
|  |  | 4-Reverse | GCCTGCTTCACCACCTTC |

**1.2 Supplementary Table S2. Information of the antibodies**

| Antibody name | Catalogue number | Specificity | Manufacturer |
| --- | --- | --- | --- |
| Anti-ERK | #16443-1-AP | Rabbit polyclonal | Proteintech Group |
| Anti-Phospho-ERK | #ab32538 | Rabbit monoclonal | Abcam |
| Anti-JNK | #ab179461 | Rabbit monoclonal | Abcam |
| Anti-Phospho-JNK | #ab124956 | Rabbit monoclonal | Abcam |
| Anti-p38 | #AF7668 | Rabbit monoclonal | Beyotime |
| Anti-Phospho-p38 | #AM063 | Mouse polyclonal | Beyotime |
| Anti-AKT | #9272 | Rabbit monoclonal | Cell Signaling Technology |
| Anti-Phospho-AKT | #sc-293125 | Mouse monoclonal | Mouse monoclonal |
| Anti-Beclin 1 | #sc-48341 | Mouse monoclonal | Mouse monoclonal |
| Anti-MAP LC3b | #sc-376404 | Mouse monoclonal | Santa Cruz |
| Anti-SQSTM1 | #sc-28359 | Mouse monoclonal | Santa Cruz |
| Anti-Bax | #2722 | Mouse monoclonal | Cell Signaling Technology |
| Anti-Bcl_2_ | #15071 | Mouse monoclonal | Cell Signaling Technology |
| Anti-RIP | #3493 | Rabbit monoclonal | Cell Signaling Technology |
| Anti-Phospho-RIP | #65746 | Rabbit monoclonal | Cell Signaling Technology |
| Anti-Caspase-3 | #14220 | Rabbit monoclonal | Cell Signaling Technology |
| Anti-Cleaved-Caspase-3 | #9664 | Rabbit monoclonal | Cell Signaling Technology |
| Anti-GAPDH | #ab9485 | Rabbit polyclonal | Abcam |
| Anti-α-Tubulin | #2125 | Rabbit monoclonal | Cell Signaling Technology |

**1.3 Supplementary Table S3. Reagents and materials**

| **Reagents** | **Source** | **Identifier** |
| --- | --- | --- |
| RAS inhibitor Abd-7 | MedChemExpress | HY-122862 |
| ERK1/2 inhibitor 1 | MedChemExpress | HY-112287 |
| PI3K-IN-30 | MedChemExpress | HY-143404 |
| AKT-IN-1 | MedChemExpress | HY-1829 |
| PKA Inhibitor Fragment (6-22) amide TFA | MedChemExpress | HY-P1290A |
| Cyclopamine | MedChemExpress | HY-17024 |
| MSAB | MedChemExpress | HY-120697 |
| Curcumin | Beyotime | SC0299 |
| Anisomycin | Beyotime | SC0132 |
| NAC | Beyotime | S0077 |
| Glycine | Beyotime | ST085 |
| T-AOC Assay Kit | Beyotime Biotechnology | S0119 |
| Reactive Oxygen Species Assay Kit | Beyotime Biotechnology | S0033S |
| CCK-8 assay kit | Beyotime Biotechnology | C0037 |
| LDH assay kit | Beyotime Biotechnology | C0016 |
| Apoptosis and Necrosis Assay Kit | Beyotime Biotechnology | C1056 |
| Senescence β-Galactosidase Staining Kit | Beyotime Biotechnology | C0602 |
| Enhanced mitochondrial membrane potential assay kit with JC-1 | Beyotime Biotechnology | C2003S |
| MitoSOX Red | MedChemExpress | HY-D1005 |
| Mito-Tracker Red CMXRos | Beyotime Biotechnology | C1035 |
| DAPI | Beyotime Biotechnology | C1002 |
| mRFP-GFP-LC3 | HanBIO Tech | HB-AP210 000 |

1. **Methods**

**2.1 Biogenesis of peptides**

All peptides (UIFSPs, N-terminal FITC-labeled UIFSP and TAT-conjugated UIFSPs) used in this work were synthesized by Sangon Biotech (Shanghai, China). HPLC and mass spectrometry were adopted to confirm purity greater than 98%.

**2.2 Peptide extraction**

Skin organisms were mixed 4 volumes of Urea Buffer (8 M urea, 1% protease inhibitor cocktail, 2 mM EDTA) for supersonic schizolysis, and the supernatants containing peptides applied to 10 kDa molecular weight cut-off (MWCO) filters. All peptide preparations were desalted with Ziptip C18 (ZTC18S960, Merck Millipore, Billerica, MA) for liquid chromatography-mass spectrometry analysis.

**2.3 Liquid chromatography tandem mass spectrometry (LC-MS/MS)**

For peptidomics analysis, peptides were dissolved in 0.1% formic acid (solvent A), directly loaded onto a home-made reversed-phase analytical column (15 cm length, 75 μm i.d.). The gradient began with an increase from 6 to 23% in solvent B (0.1% FA in 98% acetonitrile) over 26 min, 23% to 35% in 8 min and climbing to 80% in 3 min then holding at 80% for the last 3 min, all at a constant flow rate of 400 nl/min on an EASY-nLC 1000 UPLC system (Thermo Fisher Scientific, Waltham, MA).

The peptides were subjected to nano spray ionization source ionization followed by MS/MS in a Q Exactive™ HF-X (Thermo Fisher Scientific, Waltham, MA) that was coupled online to the UPLC. Intact peptides were detected at a resolution of 120,000. A speed data-dependent method was applied for the top 10 precursor ions above a threshold ion count of 2.5E5 in the MS survey scan, with 30.0 s dynamic exclusion. The electrospray voltage applied was 2.2 kV. The automatic gain control setting of 5E4 ions was used to prevent overfilling of the ion trap. For MS scans, the m/z scan range was 400 to 1800.

**2.4 Peptidomic data analysis**

The resulting MS/MS data were processed using Maxquant search engine (v.1.6.15.0). The mass tolerance for precursor ions was set as 20 ppm in First search and 5 ppm in Main search, and the mass tolerance for fragment ions was set as 0.02 Da. Carbamidomethyl on Cys was specified as fixed modification, oxidation on Met was specified as variable modifications. Label-free quantification method was LFQ, FDR was adjusted to < 1% and minimum score for peptides was set > 40.

**2.5 High-throughput mRNA-Seq and data analysis**

For transcriptome sequencing, total RNA was extracted from each sample of WS1 cells (control group or irradiation group, which with or without peptide treatment) in 24 h after UVB exposure by TRIzol reagent (Invitrogen, Carlsbad, CA). RNA sequencing was performed in the laboratory of OE Biotech (Shanghai, China). The libraries were constructed using TruSeq Stranded mRNA LTSample Prep Kit (Illumina, San Diego, CA) according to the manufacturer’s instructions. RNA libraries were controlled for quality and quantified using the Agilent 2100 Bioanalyzer (Agilent Technologies, Santa Clara, CA). Then these libraries were sequenced on the Illumina HiSeq X Ten sequencing platform and PE150bp paired-end reads were generated.

After 3’ adaptor-trimming and low-quality reads removing by Trimmomatic (version 0.36), the high-quality clean reads were aligned to the reference genome (NCBI GRCh38.p13) with hisat2 software (version 2.2.1.0). Then, FPKM value was calculated as the expression profiles of mRNA using cufflinks, and differentially expressed RNAs (DEGs) were identified using the DESeq (2012), *P*-value < 0.05 and fold Change >2 or fold Change < 0.5 was set as the threshold. GO and KEGG pathway enrichment analysis of DEGs were respectively performed using R based on the hypergeometric distribution. The raw sequencing data have been submitted to the NCBI and are accessible at GSE222414 (<https://www.ncbi.nlm.nih.gov/geo/query/acc.cgi?acc=GSE222414>). The detailed methods were described in the Supplementary materials and Methods.

**2.6 In silico analysis of** **signal peptide predictions**

SignalP-5.0 (http://www.cbs.dtu.dk/services/SignalP/) was employed to predict the signal peptide sequences and their cleavage sites in Eukarya. For this purpose, the first 70 residues from the N-terminus of each preprotein (UIFSP-1~7) were submitted to the signal peptide prediction software to calculate the cleavage probability and signal peptide likelihood.

**2.7 Confocal microscopy observation**

WS1 cells were seeded on confocal dishes (ThermoFisher, MA, USA) and treated with FITC-labeled peptides in the medium for different time points. Then, cells were stained with DAPI and Mito-Tracker Red CMXRos (Beyotime, Nantong, China), and confocal images were taken with the confocal laser scanning microscope (Carl Zeiss, Jena, Germany).

**2.8 ROS generation analysis**

DCFH-DA assay was adopted to measure the intracellular ROS level. WS1 cells were incubated with a diluted 2’,7’-dichlorofluorescin diacetate (H2DCFDA) fluorescent probe (Beyotime, Nantong, China) for 30 min and then detected by fluorescence microscope (Olympus, Tokyo, Japan) or flow cytometry (BD FACSCelesta, New York, NY).

**2.9 Antimicrobial activity assay**

The antimicrobial activity of UIFSP-5, UIFSP-6 and UIFSP-7 was tested against several pathogens, including gram-positive bacterial strains *Staphylococcus haemolyticus* (ATCC 29970) and gram-negative bacterial strains *Escherichia coli* (ATCC 25922). The minimum inhibitory concentrations (MICs) of the peptides were determined, which the peptide was serially diluted as 0.5, 1.0 and 1.5 μM. Each microbe was collected and added to 10 ml of fresh LB broth with 1% Type I agar (Hopebiol, Qingdao, China), then placed in 90-mm petri-dishes. A small hole was created in the agar after it hardened, with a 12 μl aliquot of UIFSP then added to the hole. Lastly, the microbes were incubated at 37°C for 16-18 h, with antimicrobial activity determined by the formation of a clear zone on the agar surface, which represented bacterial growth inhibition.

**2.10 Wound healing assay**

WS1 cells were treated with UIFSPs or TAT-conjugated UIFSPs for 48 h until reaching confluence. A straight wound was induced by scratching with a bacteria-free pipette and rinsing twice with PBS. Then, cells were cultured in medium without FBS. The speed of wound closure was observed and photographs were taken with a regular inverted microscope every 6 h from 0 h to 24 h. The simulated wound closure rate was calculated as follows: wound closure rate = (A_0_ − A_t_)/A_0_ × 100%, where A_0_ is the original simulated wound area and A_t_ is the simulated wound area at each time point.

**2.11 Hematoxylin and eosin (H&E) staining**

The irradiated skin tissues of mice were fixed in 4% paraformaldehyde and embedded in paraffin. Serial sections (3 μm) were adhered to positively charged slides. Then, H&E staining was performed to observe the histological features of the skin and the thickness of the epidermis and dermis.

**2.12 Real-time quantitative PCR (RT-qPCR)**

Total RNA was extracted from the WS1 cells and reverse transcribed into cDNA using an oligo(dT)12-18 primer and Superscript II reverse transcriptase (Invitrogen, Carlsbad, CA). Then, SYBR green dye One Step TB Green® PrimeScript™ RT-PCR Kit (Takara, Japan) was used for amplification of cDNA. The mRNA levels of FGF 14, TTPA, DGKG and the internal standard GAPDH were quantified by quantitative real-time PCR in a Bio-Rad iCycler RT-qPCR Detection System (Bio-Rad, Berkeley, CA). The primers for qPCR are listed in Supplementary Table S1.

**2.13 Western blotting analysis**

Cells were harvested in lysis buffer (BioTeke, Beijing, China), then subjected to SDS-PAGE. The electrophoresed samples were then transferred to PVDF membranes (Millipore, Bedford, MA). After blocking non-specific antibody binding sites on the membranes, the membranes were incubated with primary antibody, against ERK (#16443-1-AP, Proteintech, Chicago, IL), p-ERK (#ab32538, Abcam, Cambridge, MA), JNK (#ab179461, Abcam, Cambridge, MA), p-JNK (#ab124956, Abcam, Cambridge, MA), p38 (#AF7668, Beyotime, Nantong, China), p-p38, (#AM063, Beyotime, Nantong, China), α-Tubulin (#ab192314, Abcam, Cambridge, MA) and GAPDH (#ab192314, Abcam, Cambridge, MA) overnight at 4 °C. The membranes were then incubated with a horseradish peroxidase-conjugated secondary antibody (Anti-rabbit, Beyotime, #A0208) for 2 h and visualized using enhanced chemiluminescence (Beyotime). Information on the antibodies is summarized in Supplementary Table S2.

**Supplementary results**

**Supplementary Table** **S4. The upregulated peptides in UVB-irradiated frog skin**

| **Number** | **Pepetide sequence** | **Length** | **Mass** | **Leading razor protein** | **Protein description** | **Start position** | **End position** | **Ratio** | **P value** |
| --- | --- | --- | --- | --- | --- | --- | --- | --- | --- |
| 1 | SGYGGGSGFG | 10 | 844.335 | g.65324 | Keratin 8 | 63 | 72 | 1.951 | 0.046 |
| 2 | KVLKQVHPDTGIS | 13 | 1420.803 | g.33075 | Histone H2B 1.1 | 59 | 71 | 3.167 | 0.043 |
| 3 | SAKEKGKFEDMA | 12 | 1339.644 | g.46989 | High mobility group protein B1 | 53 | 64 | 1.737 | 0.039 |
| 4 | SGGYGGGSRDYYS | 13 | 1324.532 | g.73783 | Cold-inducible RNA-binding protein-like | 151 | 163 | 2.156 | 0.039 |
| 5 | AGGYDVDKNNSRLK | 14 | 1535.769 | g.34398 | Histone H1 | 83 | 96 | 1.494 | 0.038 |
| 6 | PEPAKSAPAPK | 11 | 1091.597 | g.33075 | Histone H2B 1.1 | 17 | 27 | 2.351 | 0.036 |
| 7 | SSDDVDEGARQIK | 13 | 1418.663 | g.68918 | Collagen type VI alpha 3 chain | 546 | 558 | 2.330 | 0.025 |
| 8 | SGGYGGGSRDY | 11 | 1074.436 | g.73783 | Cold-inducible RNA-binding protein-like | 151 | 161 | 1.575 | 0.016 |
| 9 | SPEGAEAKDAGKVT | 14 | 1358.667 | g.54821 | High mobility group nucleosome-binding domain-containing protein 3 isoform X2 | 6 | 19 | 2.846 | 0.009 |
| 10 | DSYDSYASNNE | 11 | 1263.452 | g.73783 | Cold-inducible RNA-binding protein-like | 180 | 190 | 1.831 | 0.007 |
| 11 | SGGGGGGGLGSGGSIRSS | 18 | 1405.654 | CON__P35527 | Undefined | 14 | 31 | 5.976 | 0.006 |
| 12 | GGYDVDKNNSRLK | 13 | 1464.732 | g.34398 | Histone H1 | 84 | 96 | 2.593 | 0.004 |
| 13 | AVAASKERSGVS | 12 | 1160.614 | g.34398 | Histone H1 | 62 | 73 | 1.816 | 0.002 |

**Supplementary Table S5. The downregulated peptides in UVB-irradiated frog skin**

| **Number** | **Sequence** | **Length** | **Mass** | **Leading razor protein** | **Protein description** | **Start position** | **End position** | **Ratio** | **P value** |
| --- | --- | --- | --- | --- | --- | --- | --- | --- | --- |
| 1 | SQSIRQTTYS | 10 | 1169.567 | g.82434 | Keratin, type I cytoskeletal 19 | 2 | 11 | 0.627 | 0.048 |
| 2 | GTKPDAPDIDLK | 12 | 1268.661 | g.46994 | PDZ domain-containing protein | 992 | 1003 | 0.608 | 0.038 |
| 3 | GPKMEGDIKGPKVD | 14 | 1469.754 | g.46994 | PDZ domain-containing protein | 1114 | 1127 | 0.592 | 0.026 |
| 4 | KTPCIIPDPCQ | 11 | 1327.626 | g.35258 | Uncharacterized protein | 62 | 72 | 0.551 | 0.014 |
| 5 | RTAVCIENSCMLR | 13 | 1608.753 | g.9716 | Postn protein | 464 | 476 | 0.403 | 0.011 |
| 6 | KTPETNKTPSAKPADLRPGDVS | 22 | 2308.202 | g.8483 | Non-muscle caldesmon | 751 | 772 | 0.341 | 0.010 |

**Supplementary Table S6. Antimicrobial activity of UIFSPs**

| **Microorganism** | **Antimicrobial Activity** | | | |
| --- | --- | --- | --- | --- |
|  | **Amoxycillin** | **UIFSP-5** | **UIFSP-6** | **UIFSP-7** |
| Gram-Positive Bacteria | | | | |
| *Staphylococcus aureus* (ATCC 29970) | + | - | - | - |
| Gram-Negative Bacteria | | | | |
| *Escherichia coli* (ATCC 25922) | + | - | - | - |

‘+’ indicates antimicrobial activity; ‘-’ indicates no antimicrobial activity


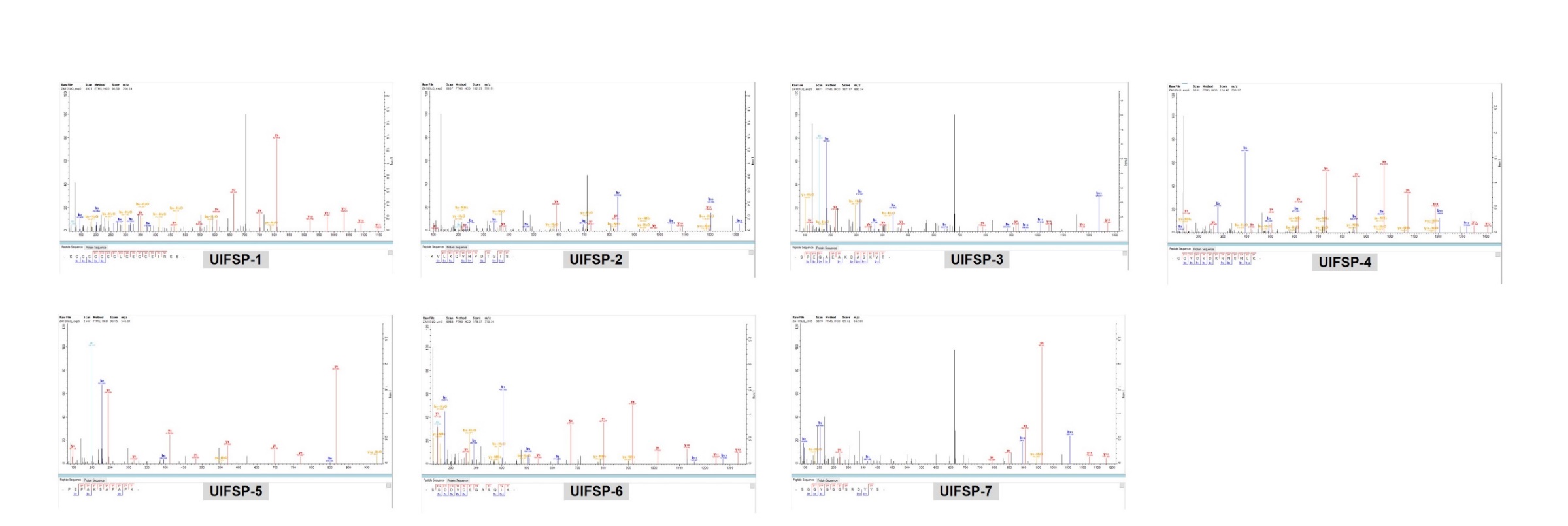


**Supplementary Fig. S1. MS/MS annotation of UIFSP-1~7 from *P. nigromaculatus* skin.**
Amino acid sequence of UIFSP-1 was SGGGGGGGLGSGGSIRSS, UIFSP-2 KVLKQVHPDTGIS, UIFSP-3 SPEGAEAKDAGKVT, UIFSP-4 GGYDVDKNNSRLK, UIFSP-5 PEPAKSAPAPK, UIFSP-6 SSDDVDEGARQIK, UIFSP-7 SGGYGGGSRDYYS.


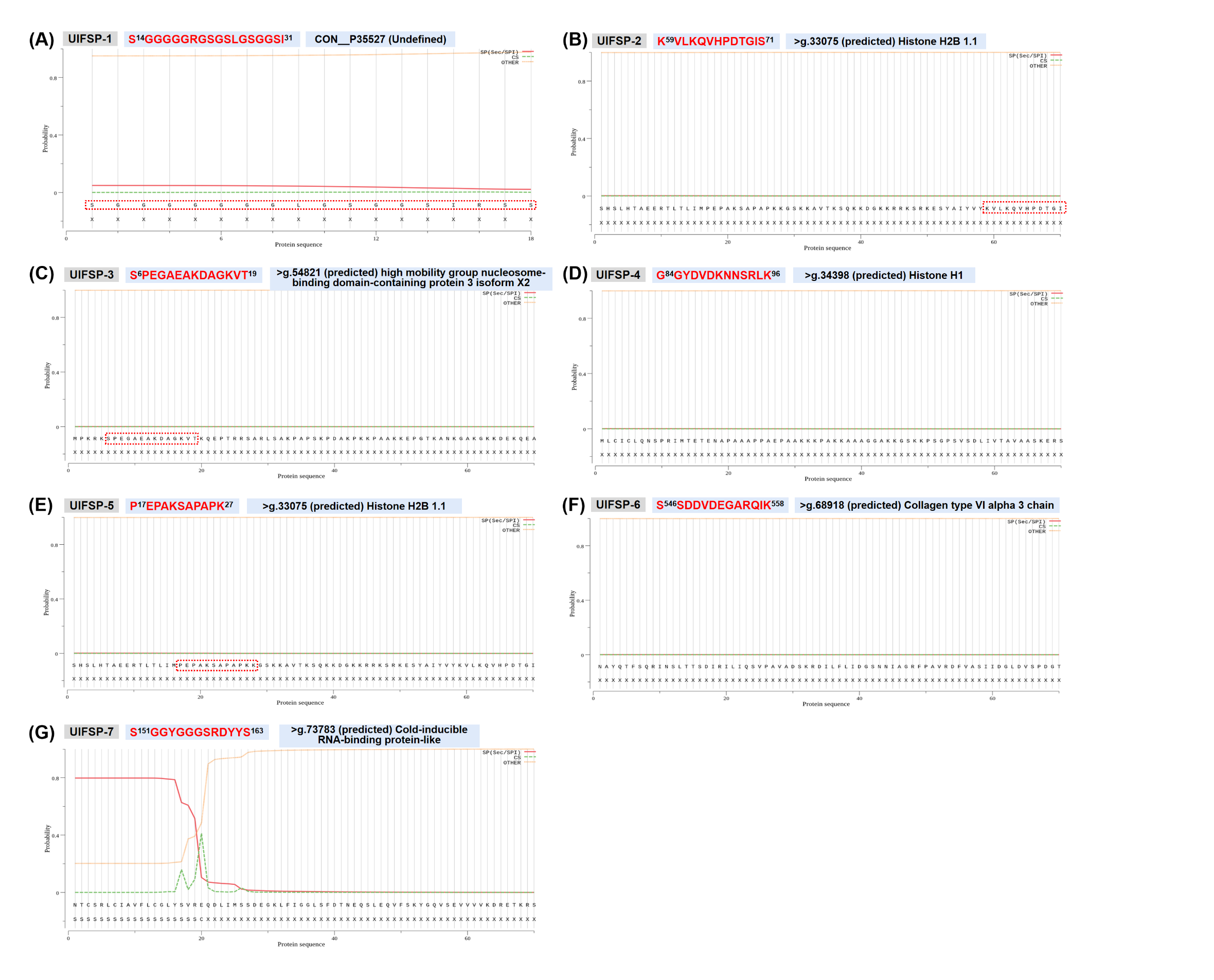


**Supplementary Fig. S2.** Prediction of the signal peptide derived from UIFSPs preproteins using SignalP-5.0. (A) UIFSP-1, (B) UIFSP-2, (C) UIFSP-3, (D) UIFSP-4, (E) UIFSP-5, (F) UIFSP-6, and (G) UIFSP-7 preprotein. The first 70 residues from N-terminus of the each preprotein were selected for signal peptide prediction.


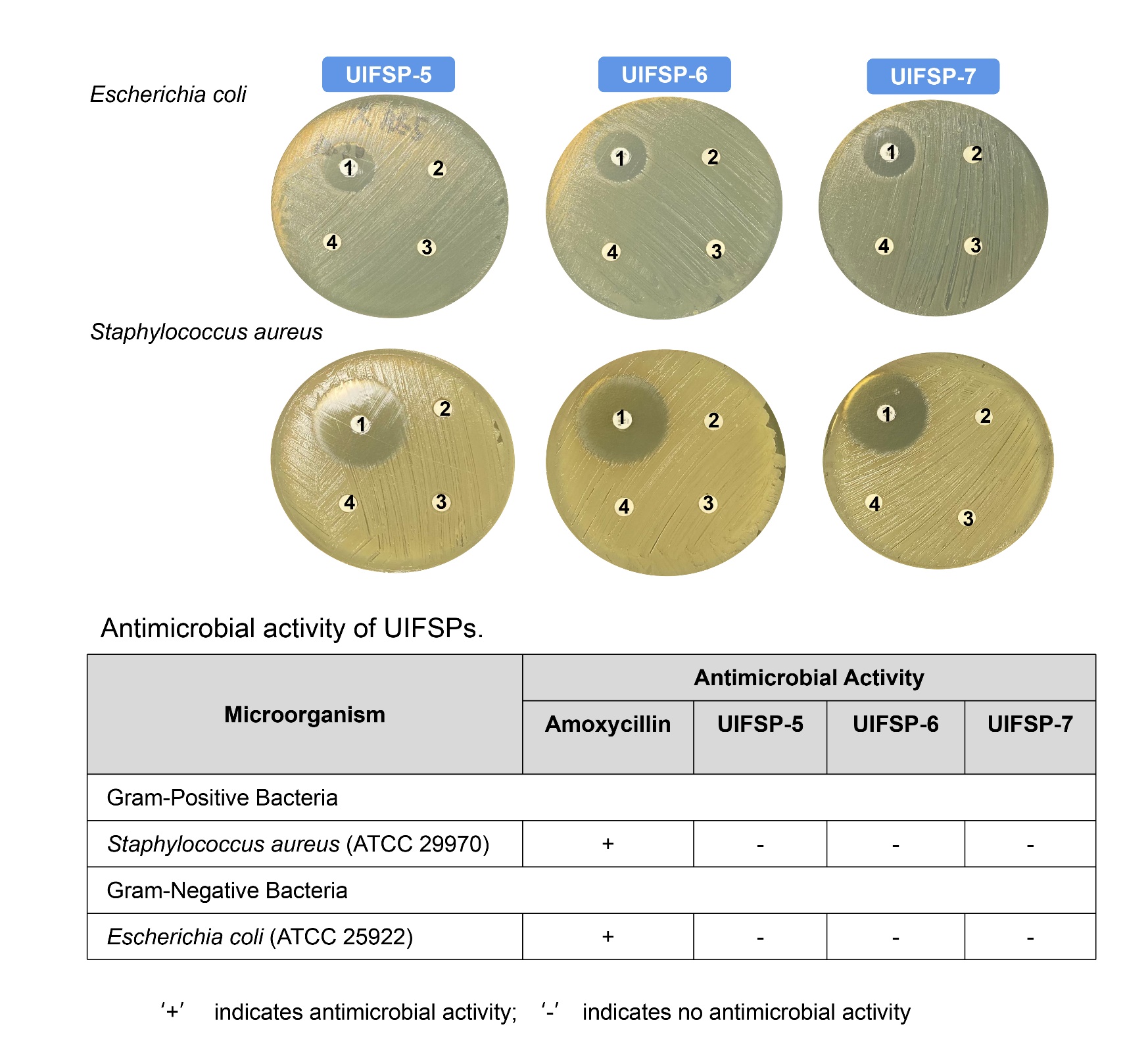


**Supplementary Fig. S3. UIFSPs show no antimicrobial activity.**
The antimicrobial activity of UIFSP-5, UIFSP-6 and UIFSP-7 was tested against *Staphylococcus aureus* (gram-positive bacterial strain) and *Escherichia coli* (gram-negative bacterial strain). No. 1, positive control (amoxycillin, 2.0 μg); No. 2, UIFSP peptide (1.5 mM); No. 3, UIFSP peptide (1.0 mM); No. 4, UIFSP peptide (0.5 mM).


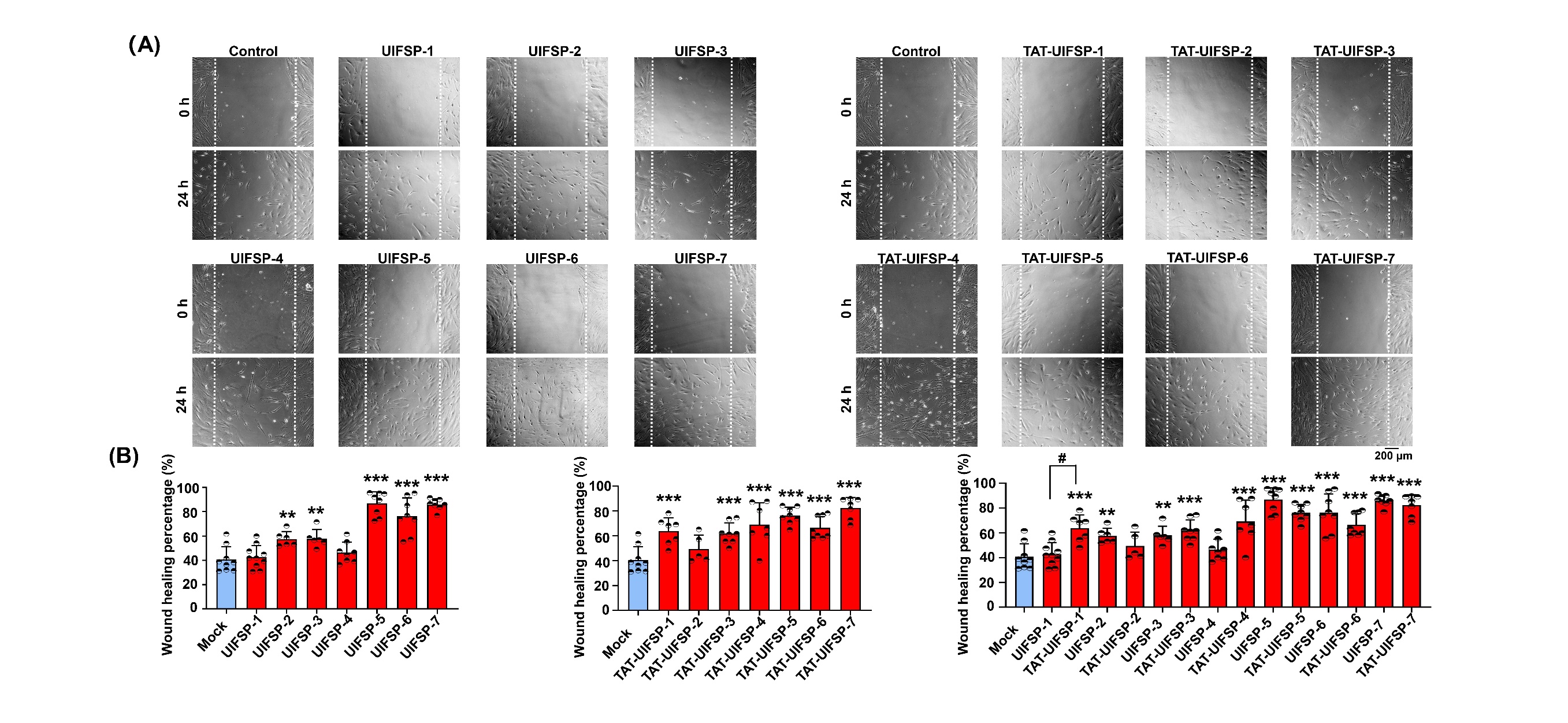
**Supplementary Fig. S4. UIFSPs promote cell migration.**

(A) and (B) Representative images and quantitation of migrated cells measured by a scratch wound model in vitro. WS1 cells were pretreated with UIFSPs or TAT-conjugated UIFSPs (TAT-UIFSP) (20 μM) for 48 h, cells were scratched to make straight wounds, and photographs were taken every 6 h from 0 h to 24 h. The results are expressed as a percentage of the initial wound area. ***P* < 0.01 and ****P* < 0.001, compared to the vehicle group. *^#^P* < 0.05, statistical difference between UIFSP and TAT conjugated UIFSP group.


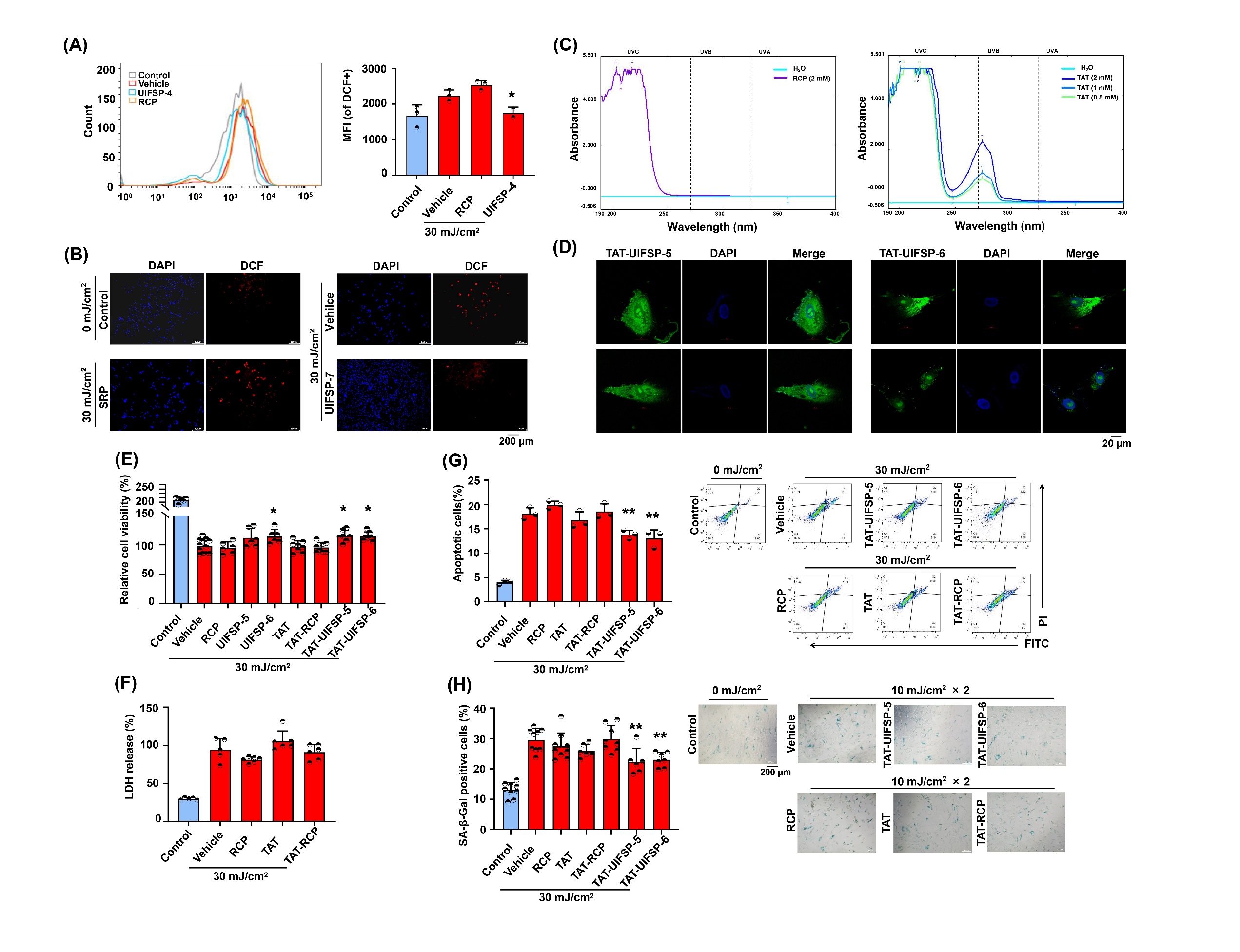


**Supplementary Fig. S5. Randomized control peptide (RCP) and TAT sequences show no protective effects in UVB-irradiated skin cells.**

(A) and (B) ROS levels in WS1 cells through flow cytometry and fluorescence. (C) Absorption spectrum in the ultraviolet region of RCP and TAT solution. RCP and TAT were dissolved in deionized water to measure the absorption spectra between 190 and 400 nm. (D) The membrane impermeability of TAT conjugated UIFSPs to WS1 cells through confocal microscopy. WS1 cells were treated with FITC-labeled TAT conjugated UIFSP-5 or -6 at a concentration of 20 μM for 24 h. Nuclei (blue) were stained with the DNA-binding dye DAPI. Scale bars, 20 μm. (E) Viability, (F) LDH activities, (G) apoptosis rate, and (H) senescence of WS1 cells, as detected by CCK-8-based assay, LDH release assay, AV/PI staining and SA-β-Gal staining analysis, respectively. WS1 cells were pretreated with RCP, TAT or TAT-conjugated UIFSPs (TAT-UIFSP-5 or -6) (20 μM) for 48 h, exposed to 30 mJ/cm2 UVB once or 10 mJ/cm2 UVB twice, and detected 36 h (WS1), 24 h or 72 h later. Student’s t test and one-way ANOVA were used to evaluate the differences between groups. **P* < 0.05, ***P* < 0.01, compared to the vehicle group.


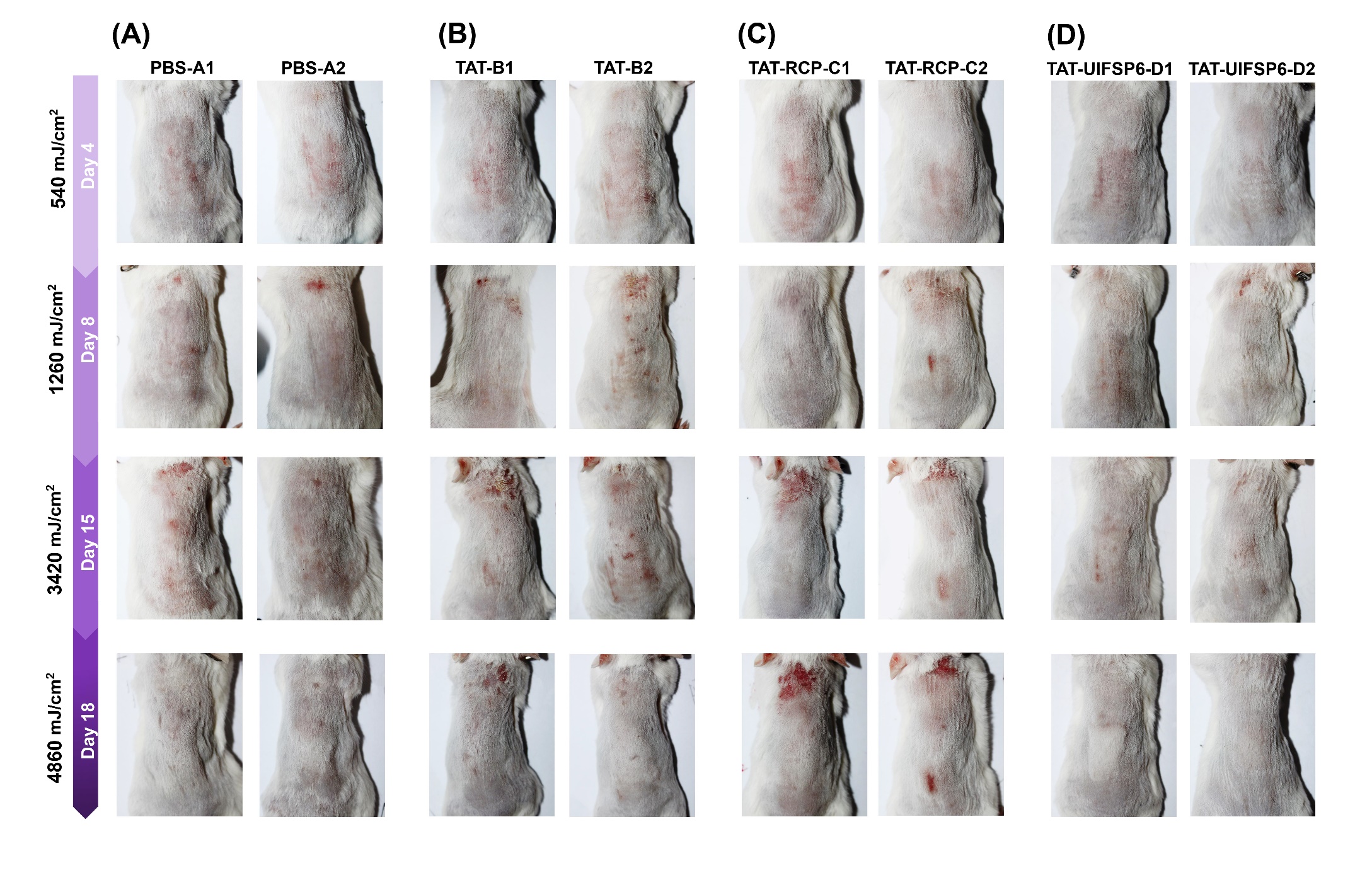
**Supplementary Fig. S6. TAT sequences show no protective effects in UVB-irradiated mice skin.**

Macroscopic changes in the dorsal skin on Day 4, 8, 15, and 18 during UVB exposure from PBS group (A), TAT group (A), TAT conjugated RCP group (C) and TAT conjugated UIFSP group (D). UVB-induced skin injury modeling was consistent with the schematic diagram of the time course in Fig 5B. During this period, 20 μM TAT-conjugated UIFSP-3, -4, -5, -6 (TAT-UIFSP-3, -4, -5 and -6) or PBS was smeared on the irradiated area after UVB radiation.


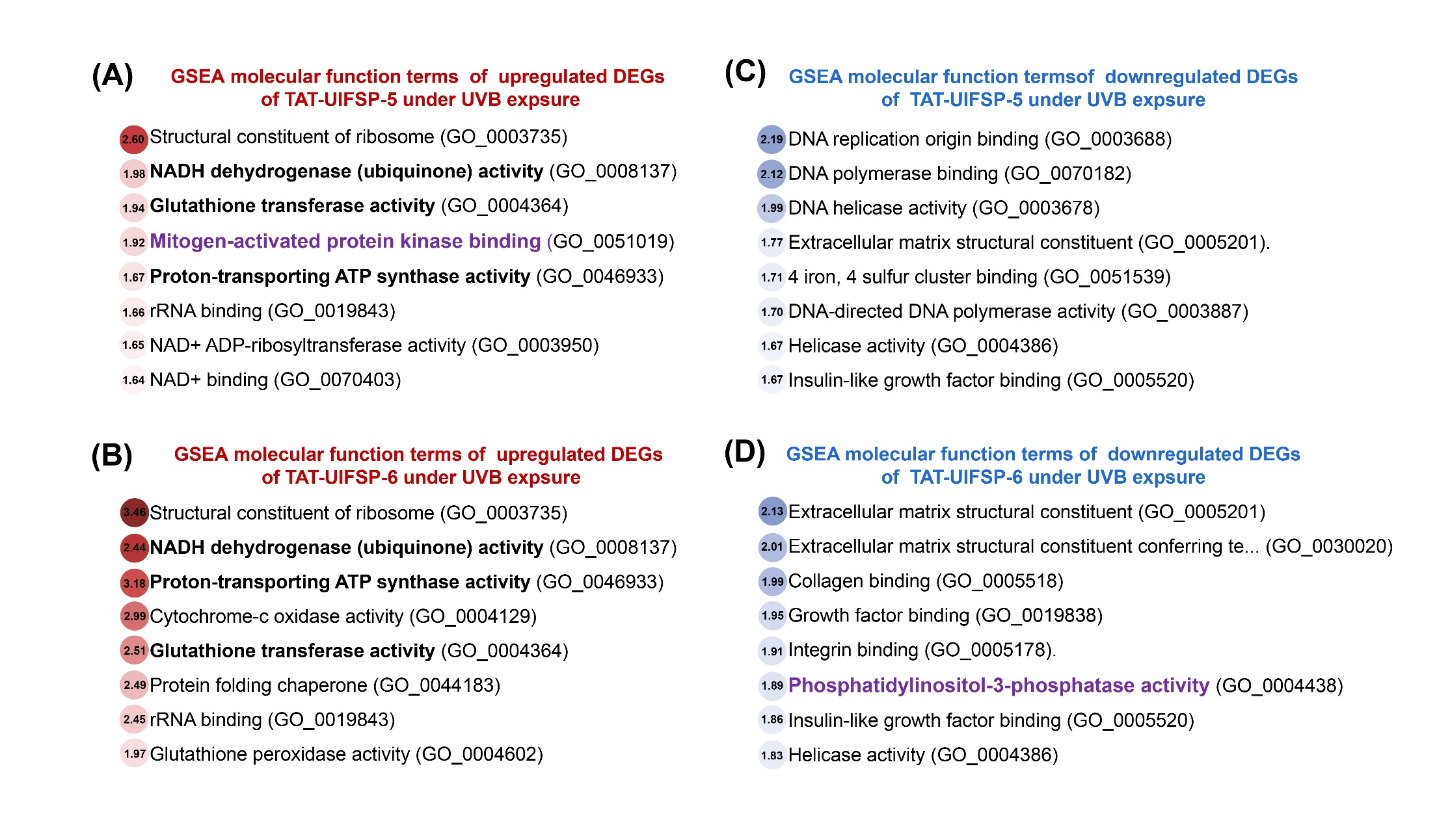


**Supplementary Fig. S7. Top GO terms (MF) of TAT-conjugated UIFSP-5 or -6 by GSEA.**

(A) and (B) Representative GO molecular function (MF) terms and pathways in TAT-conjugated UIFSP-5 group, (C) and (D) Representative GO terms and pathways in TAT-conjugated UIFSP-6 group. The color keys from white to red or blue indicate the enrichment levels (Normalized Enrichment Score, NES) from low to high. WS1 cells were pretreated with TAT-conjugated UIFSP-5 or -6 (20 μM) for 48 h, exposed to 30 mJ/cm^2^ UVB, and collected for RNA-Seq after 24 h.


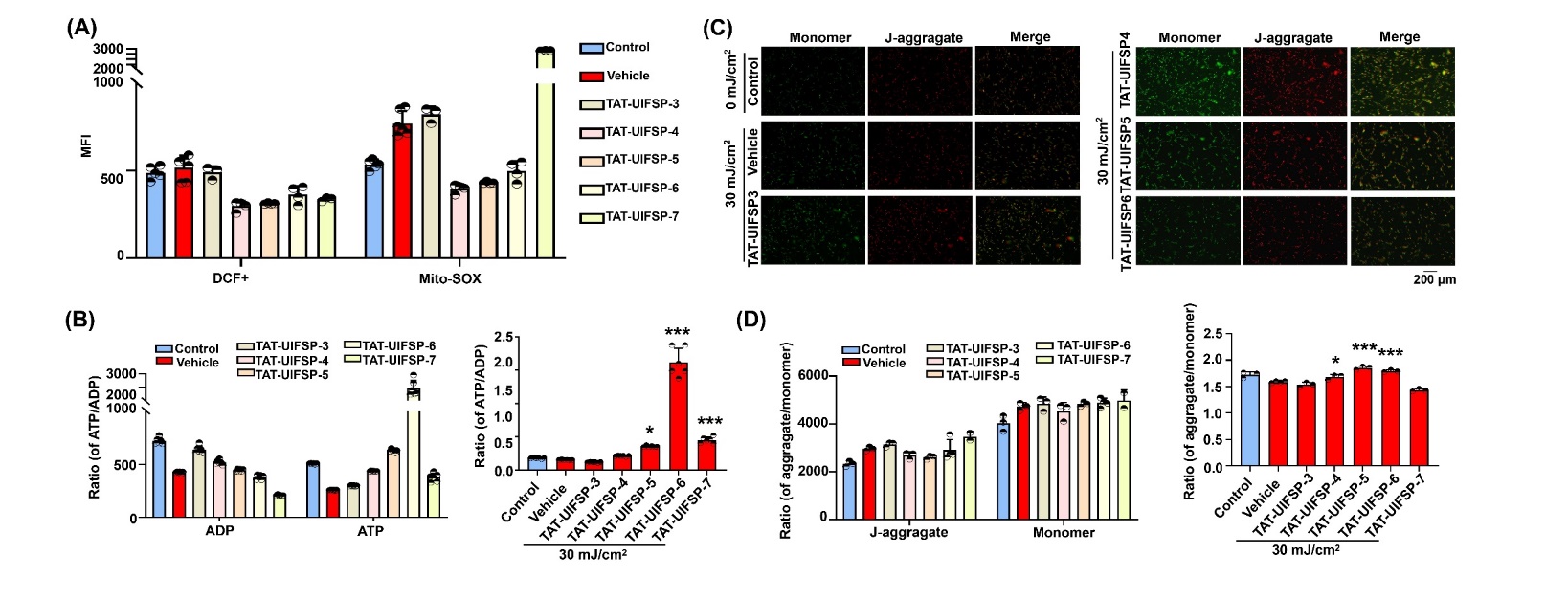
**Supplementary Fig. S8. UIFSPs alleviate UVB-induced mitochondria damage in skin cells.**

(A) Representative flowcytometry results and comparison of total ROS and mitochondrial ROS (MitoSOX) production in WS1 cells. (B) Representative flow cytometry results for ATP production in WS1 cells detected by ADP probe staining. (C) and (D) mitochondrial membrane potential (MMP) in WS1 cells through fluorescence and flow cytometry by JC-1 staining. Skin cells were pretreated with UIFSPs (20 μM) for 48 h, exposed to 30 mJ/cm^2^ UVB, then detected the indicators of mitochondria damage 36 h later. Student’s *t* test and one-way ANOVA were used to evaluate the differences between groups. **P* < 0.05 and ****P* < 0.001, compared to the vehicle group.


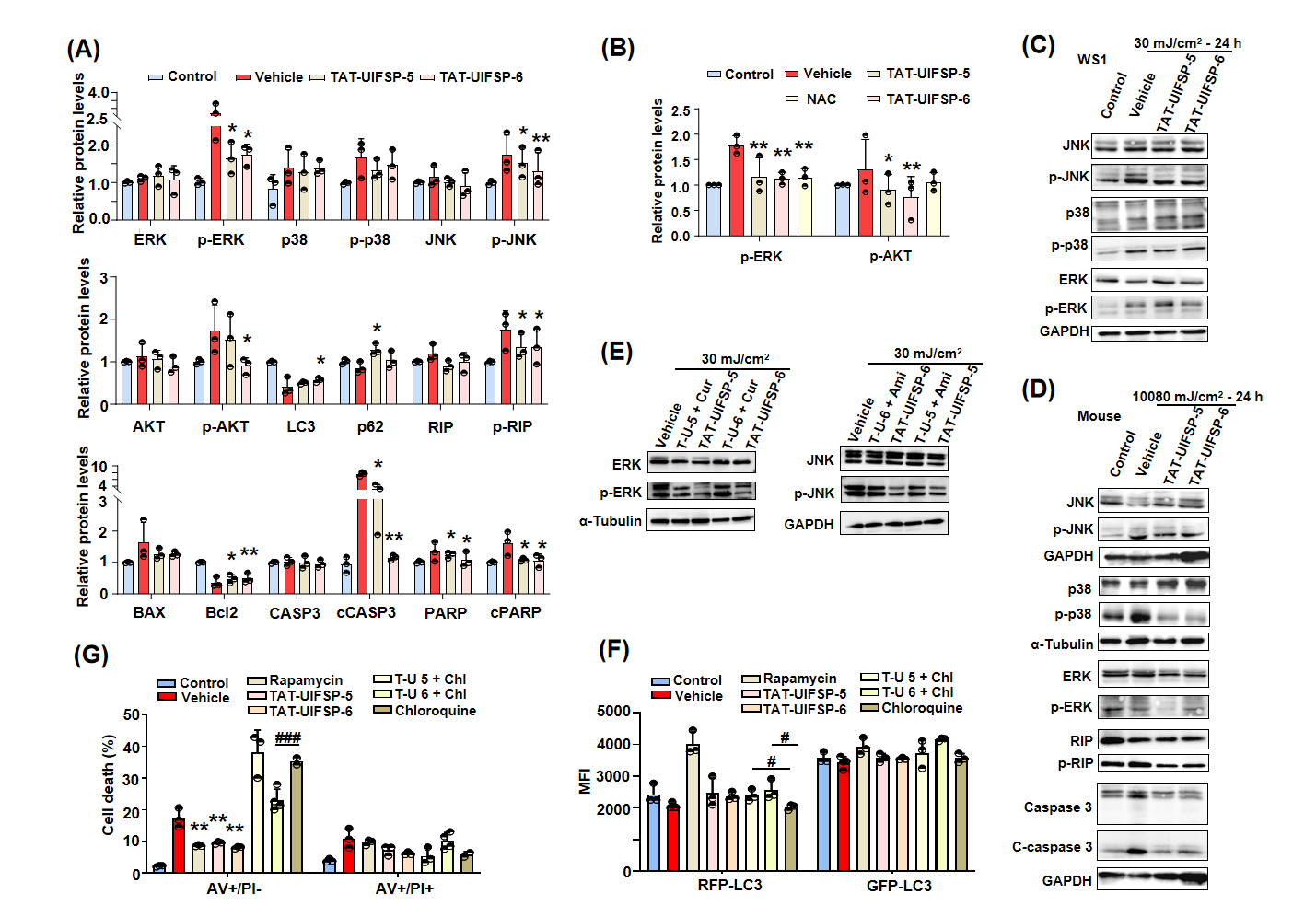


**Supplementary Fig. S9. UIFSPs** **inhibit the activation of MAPKs and stimulate cell autophagy in UVB-exposed cells.**

(A) and (B) Expression levels of protein blots in FIGURE 7E and 7F were quantified by ImageJ image processing program, *n*=3. (C), (D) and (E) Western blots showing the change in the expression of protein markers related to the MAPK signaling pathway and cell death in WS1 cells and mice. Western blotting analysis were performed for at least three independent experiments. Cells and mice were pretreated with TAT-conjugated UIFSP-5 or -6 (TAT-UIFSP-5 or -6) or peptide in combination with anisomycin (1 μM) or curcumin (20 μM), then exposed to UVB (30 mJ/cm^2^ or 10080 mJ/cm^2^) and detected 24 h or 36 h later. Flow cytometry analysis of (D) autophagy and (E) apoptosis by mRFP-GFP-LC3 probe and AV/PI staining in irradiated WS1 cells pretreated with TAT-conjugated UIFSP-5 or -6 (20 μM). Student’s *t* test and one-way ANOVA were used to evaluate the differences between groups. ***P* < 0.01, compared to the vehicle group. *^#^P* < 0.05, *^###^P* < 0.001, statistical difference between two specified groups.
